# Impact of adaptive filtering on power and false discovery rate in RNA-seq experiments

**DOI:** 10.1186/s12859-022-04928-z

**Published:** 2022-09-24

**Authors:** Sonja Zehetmayer, Martin Posch, Alexandra Graf

**Affiliations:** Center for Medical Statistics, Informatics, and Intelligent Systems, Medical University of Vienna, Spitalgasse, Vienna, Austria

**Keywords:** Next generation sequencing, Gene expression, Multiple testing, Gene filter

## Abstract

**Background:**

In RNA-sequencing studies a large number of hypothesis tests are performed to compare the differential expression of genes between several conditions. Filtering has been proposed to remove candidate genes with a low expression level which may not be relevant and have little or no chance of showing a difference between conditions. This step may reduce the multiple testing burden and increase power.

**Results:**

We show in a simulation study that filtering can lead to some increase in power for RNA-sequencing data, too aggressive filtering, however, can lead to a decline. No uniformly optimal filter in terms of power exists. Depending on the scenario different filters may be optimal. We propose an adaptive filtering strategy which selects one of several filters to maximise the number of rejections. No additional adjustment for multiplicity has to be included, but a rule has to be considered if the number of rejections is too small.

**Conclusions:**

For a large range of simulation scenarios, the adaptive filter maximises the power while the simulated False Discovery Rate is bounded by the pre-defined significance level. Using the adaptive filter, it is not necessary to pre-specify a single individual filtering method optimised for a specific scenario.

**Supplementary Information:**

The online version contains supplementary material available at 10.1186/s12859-022-04928-z.

## Background

In next generation RNA-sequencing experiments (RNA-seq), thousands of genomic features (typically genes) are investigated to study differential expression levels among several experimental conditions. Often the number of replicates per condition is small. When testing each gene individually, multiple testing procedures have to be applied to avoid an increase in false positive results and the power to detect truly differentially expressed (DE) genes between conditions is often low.

It has been shown that data filtering can increase the number of rejections or the power, respectively, of high-throughput experiments [1–3]. Filtering removes genes with, e.g., low counts or small variation in the pooled sample, or genes whose counts are so poorly measured, that their expression level cannot be determined. In RNA-seq, genes with low counts across all replicates or samples (also called libraries) are assumed to provide little evidence for differential expression as a gene must be expressed at some minimum level before it is likely to be transformed to a protein or to be biologically important [4]. On the other hand, genes with low variation in the pooled sample and which therefore have small chance of showing a significant differential expression in the final analysis, are removed. Only genes that pass a pre-specified filter are part of the final analysis and are considered in the multiple testing procedure. With a lower number of genes, a less stringent multiplicity adjustment can be applied and thus the probability to detect truly DE genes is increased.

Several methods for filtering were proposed for RNA-seq data, e.g., filtering based on the maximum or the mean of the observed counts across all groups (e.g., treatment conditions) for each gene, with varying thresholds for the selection. It has been shown that filtering should be based on blinded data, i.e., independent of the groups, to avoid inflation of the Type I error rate (for details, see [5]). However, there is no consensus or rule on the choice of the filtering process or the threshold values. E.g., the vignette of the bioconductor package EdgeR [4] states: ’As a rule of thumb, genes are kept if they are expressed in at least one condition. Users can set their own definition of genes being expressed.’ Thus, in practice, a user may apply various filtering strategies and chose the rule leading to the ’best’ result for the final analysis. However, it has not been investigated, how this approach affects the Type I error rate and the power.

In this paper, we propose an adaptive filter for RNA-seq data which selects one out of several filtering methods aimed at maximising the number of rejections. In a large range of simulation scenarios, practically no impact on the False Discovery Rate (FDR) is observed, if several filters are considered and the filter leading to the largest number of rejections is selected. No additional adjustment for multiplicity for the number of considered filters has to be included. The only additional rule that has to be considered is that if the number of rejections is too small (and lies below a specified filter parameter), a previously defined reference filter has to be chosen for the final analysis. Otherwise the FDR may be inflated. In the next section, we show that no uniformly optimal filter in terms of multiple power exists and that the proposed adaptive filter leads to the largest power without inflation of the FDR for many different simulation scenarios. The merits of the new adaptive filter procedure are illustrated using simulated and real data. In the Methods section, we review the data processing steps for RNA-seq data and define the adaptive filter.

## Results

We first perform simulation studies to compare filtering methods from the literature with regard to multiple power, defined as the proportion of correctly rejected false null hypotheses under all false null hypotheses, i.e. the proportion of correctly identified truly DE genes under all DE genes (hereinafter denoted as power). Since there is no consensus rule on the choice of the filtering process, we propose the new adaptive filter approach which searches for the best filter in terms of the number of identified genes (as described in more detail in the Methods section). We then analyse the impact of the adaptive filter on the multiple power and the FDR.

**Table 1:** Simulation strategies. More details for each setting can be found in the Additional file 1

Simulation	Description and data sources
NB	The count data are assumed to follow a negative binomial distribution (NB), dispersion and mean parameters are fixed and equal for all $H_0$ or $H_1$, respectively.
NB with distributed parameters	Read counts follow a NB distribution, dispersion and mean parameters vary across genes and are based on real RNA-seq data sets according to [2] (real data sets Kidney [6], Bottomly [7], and Sultan [8], see Table 3).
SimSeq [9]	Counts based on real data read counts adjusted by a correction factor to generate differential expressions, dependence between genes is imitated from real data sets Bottomly [7], Kidney [6], and mouse [10].
PROPER [11]	Read counts follow a NB distribution, dispersion and mean parameters vary across genes and are based on a real RNA-seq data set (Cheung [12]). Additional noise is introduced due to zero baseline expressions in the original data leading to many genes with zero counts only.
PROPER with fixed sequencing depth [11]	As PROPER. Here, the empirical average expressions sampled from the Cheung data are standardised to reach a fixed sequencing depth.

### Simulation settings

In the simulation study, we consider experiments comparing two groups of independent samples with $m=10000$ two-sided null hypotheses $H_{0i}$, $i=1,\dots ,m$ (corresponding to genes or features, hereinafter referred to as genes). The two groups are of sizes $n_1=n_2=10$, and we consider different proportions $\pi _0$ of true null hypotheses, i.e. non-DE genes, in the range from 0.5 to 1. To simulate RNA-seq data, we consider a wide range of strategies (see Table 1 and Additional file 1).

**Table 2:** Types of filtering methods

Filter	Description	Considered thresholds
Mean-based	These filters are based on the gene-wise overall mean counts from both conditions. Genes with a mean expression less than some threshold given by the specified percentile percentage of mean counts are removed by the filter and not considered for the test decision (e.g., [2]).	Percentile % $=\{$1, 2, 3, 4, 5, 8, 11, 14, 17, 20, 23, 26, 29, 32, 35, 38, 41, 44, 47, 50, 55, 60, 65, 70,75, 80, 85, 90$\}$
Max-based	Genes with maximum counts (over both conditions ) less than a threshold given by the specified percentile percentage of maximum counts are removed from the analysis and not considered for the test decision (e.g., [2]).	Percentile % $=\{$1, 2, 3, 4, 5, 8, 11, 14, 17, 20, 23, 26, 29, 32, 35, 38, 41, 44, 47, 50, 55, 60, 65, 70, 75, 80, 85, 90$\}$
CPM	Robinson and Oshlack (2010) [13] propose to base filtering on counts per million (CPM). Genes with CPM values less than threshold *c* in more than $\min (n_1,n_2)$ samples are removed.	$c=\{1,2,5,25,50,100\}$
Jaccard	Max-based filter [2] where the filter threshold $v^*$ is determined with the Jaccard similarity index. To compute the index for a pair of replicates, the gene counts are first dichotomised for a cut-off *v*: a gene count is either larger than *v* or not. Then the number of counts larger than threshold *v* in *both* replicates divided by the number of gene counts larger in *any* of the two replicates is calculated resulting in values between 0 (dissimilar) and 1 (similar). The global Jaccard index is the average of the index across all pairs in each condition. The calculations are repeated for several threshold values *v* and the threshold $v^*$ with the greatest similarity is found by fitting a loess curve through the set of candidate thresholds. $v^*$ is then used as a threshold in a max-based filter.	
Zero-based	This filter counts the sum of zero counts per gene and removes genes with more than *u* zeros from the analysis. Note that the basic filter is the zero-based filter with threshold $u=n$.	$u=\{16,\dots , 1\}$

**Fig. 1:**
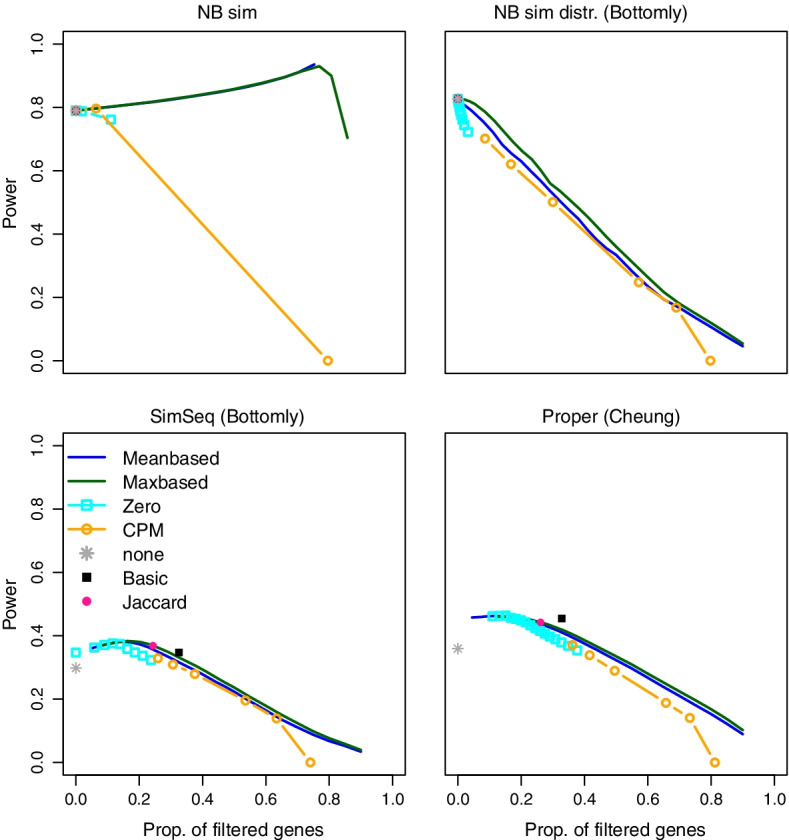
**Power comparison of different filters.** Power values for several filtering methods and simulation strategies for $\alpha =0.05$, $\pi _0=0.8$, $m=10000$, $n_1=n_2=10$ (or $n_1=n2=5$ for SimSeq (Bottomly)). The power of each filtering method is plotted as a function of the actual mean proportion of filtered genes across all simulation runs for the set of genes with at least one non-zero count; only the proportion of the basic filter is based on the total number of hypotheses *m*. The basic, Jaccard and no filter results are represented by a point because these methods are based on a fixed threshold

**Fig. 2:**
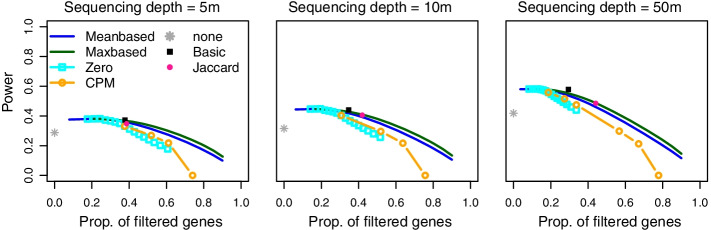
**Power comparison of different filters and sequencing depths.** Power values for several filtering methods for PROPER simulation for $\alpha =0.05$, $\pi _0 =0.8$, $m=10000$, $n_1=n_2=10$ and sequencing depths 5*m*, 10*m*, and 50*m*. The power of each filtering method is plotted as a function of the actual mean proportion of filtered genes across all simulation runs for the set of genes with at least one non-zero count; only the proportion of the basic filter is based on the total number of hypotheses *m*. The basic, Jaccard and no filter results are represented by a point because these methods are based on a fixed threshold

Filtering was performed in two steps: We first filtered the raw counts and removed genes with only zero counts in both groups. This filter is denoted as the basic filter. Based on the remaining genes, the data were normalised and for each gene a p-value for group comparison was calculated (see Methods section). Next, one of the filters listed in Table 2 was applied to the raw counts. The unadjusted p-values of the hypotheses selected by the two filters (the basic filter and the chosen additional filter) were then adjusted for multiplicity. In our simulations we examined and discussed two methods for multiplicity adjustment, the local False Discovery Rates (lfdr) [14, 15] and the Benjamini-Hochberg (BH) procedure [16]. The significance threshold $\alpha$ was set to 0.05 (for alternative analysis pipelines, see the Methods and the additional files). For the adaptive filter strategy, all filters in Table 2 are applied separately and the method that leads to the largest number of rejections is selected as the actual filter. Note that in the simulations we also considered the options to perform no filtering at all and the option to perform no further filtering step after the basic filter (these options are in the following referred to as ’none’ and ’basic’). The considered filters (Table 2) are parameterised by thresholds with the exception of the Jaccard filter, which has no parameter. For each of these filters we considered a range of thresholds. Such thresholds can be specified as absolute constants (e.g. a minimum mean count) or as percentile percentages (e.g. the 5% percentile of the observed mean counts). For mean and max-based filtering, we chose thresholds based on percentile percentages and for CPM and zero-based filtering a fixed set of thresholds is used. For the comparisons in Figs. 1 and  2, the corresponding proportions of filtered genes (i.e. removed genes) in the respective scenario are plotted and computed after removing genes with only zero counts. Note that also for methods based on percentiles percentages, the actually observed proportions plotted in the Figures may differ from the nominal percentages due to ties.

All simulations were performed with R version 3.6.0 or higher [17]. At least 1000 simulation runs were performed for each scenario.

### Comparison of individual filtering strategies

We first compare the individual filtering methods with regard to the power. A comparison with the adaptive filtering approach is reported in the next section.

For several simulation settings, the power of each filter as a function of the proportions (across all simulation runs) of filtered genes among the set of genes selected by the basic filter is shown in Figs. 1 and 2 for the lfdr adjustment and $\pi _0=0.8$. The corresponding results for $\pi _0=0.5$ and 0.99, simulations based on other real data sets, and for the BH procedure can be found in Additional file 2: Figs. S1, S2, S13–S15. For a better comparison of filtering methods in the plots, the thresholds are transformed to the corresponding proportions of filtered genes. Only the proportion of selected genes reported for the basic filter is based on the total number of hypotheses *m*, the proportion of no filtering is always 0.

It can be seen that in many scenarios mean and max-based filters maximise the power, however, the optimal percentile percentage depends very sensitively on $\pi _0$, the simulation setting and the multiplicity adjustment. Often the optimal filter is not unique, as several methods/percentile percentages lead to the same or very similar power values. For example, for NB data simulation more than half of the genes should be removed to maximise power and the larger $\pi _0$, the more genes should be removed by the filter. For the NB simulation with distributed dispersion and mean parameters, however, optimal power values are achieved for much lower percentile percentages. The basic filter (which in this simulation setting is equivalent to no filtering as zero counts in the whole sample are very unlikely) as well as all other filters with very low percentile percentages show a good performance in all considered scenarios. In other scenarios, e.g., SimSeq simulations with counts based on the mouse or Bottomly data sets, the max and mean-based filters have higher power values if the percentile percentage of filtered genes is approx. between 10% and 20%. However, the advantage in terms of power is only small (between 0.3 and 0.6 percentage points). In contrast, for the SimSeq simulation based on the Kidney data set, hardly any genes should be filtered (see Fig. 1 and in Additional file 2: Figs. S1, S2, S13–S15). Note that, as expected, the resulting power of the BH adjusted simulations is larger. Apart from that the curves look rather similar and optimal percentile percentages are comparable.

The PROPER simulations show rather flat power curves near the optimum, the power is increased by 0.5 to 2 percentage points in comparison to the basic filter. As this simulation generates many zero genes, the advantage of the basic filter compared to no filtering is very pronounced. The optimal power is observed with the max-based and the zero filter and lies between the 10% (for $\pi _0=0.5$) and 20% percentile (for $\pi _0=0.99$). Figure 2 shows the results of the simulation study with fixed sequencing depths = 5*m*, 10*m*, and 50*m* for the lfdr method. As expected, power increases with sequencing depth. Optimal power values can be found for the zero and the max-based filter, the optimal percentile percentage decreases for an increasing sequencing depth from 0.24 to 0.12.

For the simplistic simulation settings such as NB sim and NB sim distributed, the ’none’ filter often shows similar power values as the basic filter. However, for the SimSeq and the PROPER simulations, large power increases of more than 20% can be found. Moreover, as can be seen in Additional file 2: Figs. S4–S7 and 17-19, in many scenarios the FDR of the ’none’ filter option is increased.

None of the filters is uniformly optimal across all considered scenarios, however, in many scenarios, mean and max-based filters with low thresholds, zero filter with high thresholds, the Jaccard or the basic filter generate the highest power values. However, huge differences in power of more than 60% can be observed if inefficient thresholds are chosen. Note again, that the optimal filter and/or threshold is unknown in practice.

A modified order of data processing, where filtering was performed before normalisation and data analysis (order (b)) as described in the Methods section), can be found in the Additional file 2: Figs. S22 and S23 (multiplicity adjustment with lfdrs). The results emphasise the conclusion that the optimal filter depends on the simulation scenario.

### Adaptive filter

Since none of the considered filters is uniformly optimal, the adaptive filter may be a useful option to avoid the risk of choosing an inefficient filter in terms of power. In this section, the simulation studies presented above are extended and the performance of the adaptive filter is investigated. The adaptive filter incorporates all filters as described in Table 2 and finally applies the filter which leads to the largest number of rejections. To avoid an increase in the FDR under the global null hypothesis where $\pi _0=1$, a filter parameter *l* was introduced and a reference filter was defined in advance. *l* was set to 5, i.e. in case of 5 or less rejections, the reference filter and not the adaptive filter is applied. Otherwise an inflation of the FDR under the global null hypothesis may be observed. In our simulation study, the Jaccard filter was specified as the reference filter.

**Fig. 3:**
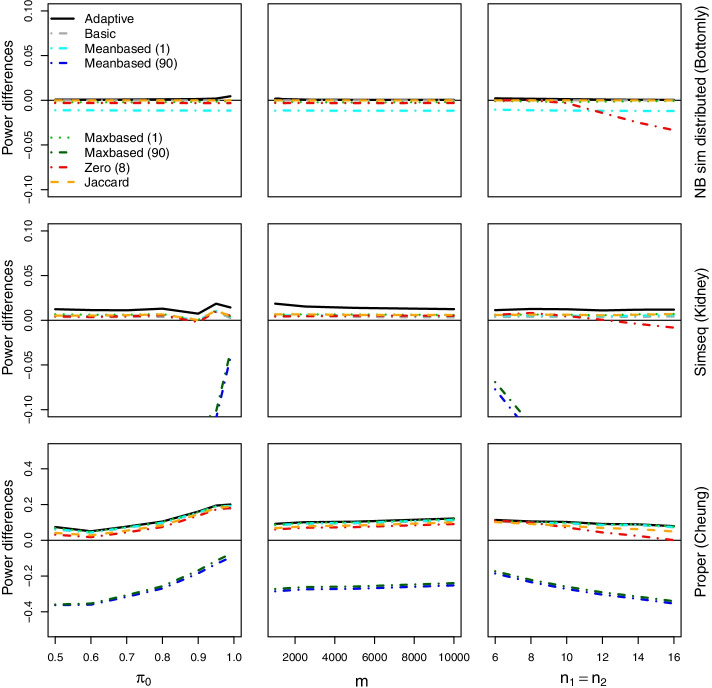
**Adaptive filter I.** Differences in power for adaptive filter and selection of applied filters compared to no filter for several scenarios. The plotted filtering methods and the corresponding percentile percentages are given in the legend. $\pi _0=0.8$, $m=10000$, $n_1=n_2=10$ or $\pi _0$, *m*, and $n_1=n_2$ are parameters on the x-axis, $\alpha =0.05$ (lfdr adjustment). Note that the range of the y-axis is chosen result-based; filtering methods with low power may not be visible on some plots

**Fig. 4:**
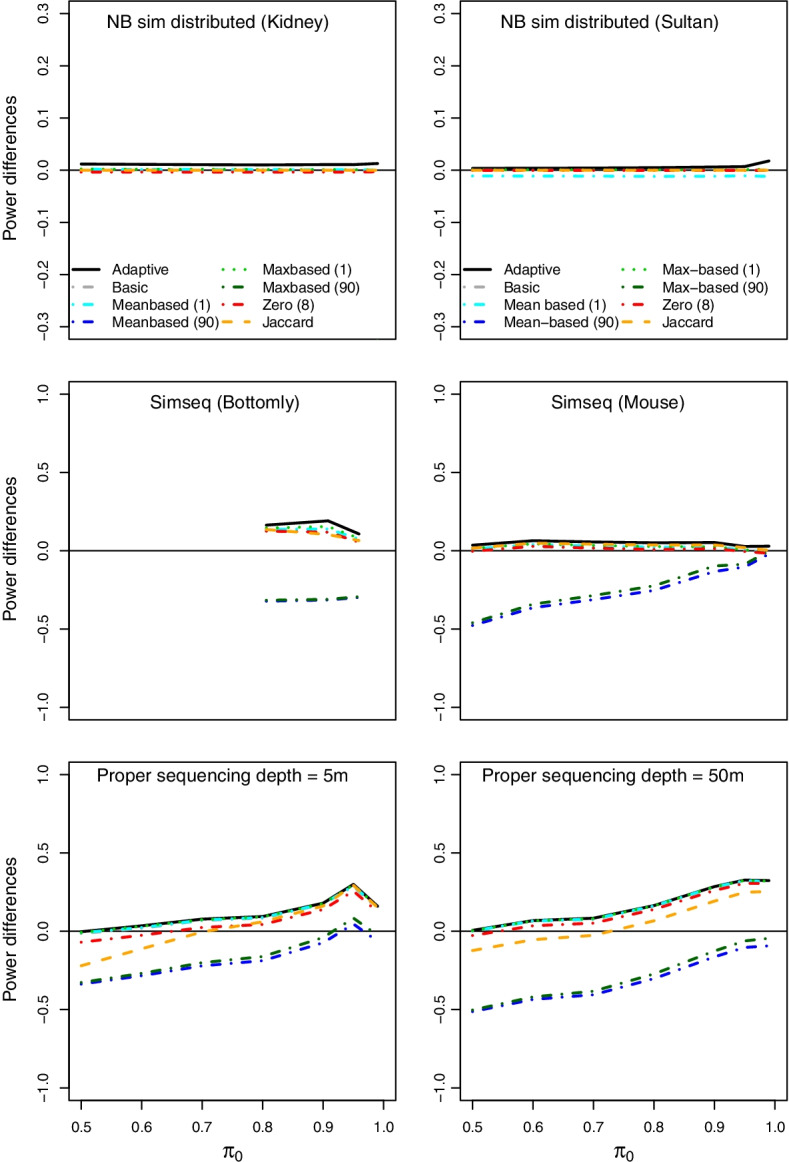
**Adaptive filter II.** Differences in power for adaptive filter and selection of applied filters compared to no filter for NB sim distributed for Kidney and Sultan data, SimSeq simulation for Bottomly and mouse mammary data sets and PROPER simulation for sequencing depths 5*m* and 50*m* for varying $\pi _0$, $m=10000$, $n_1=n_2=10$ ($n_1=n_2=5$ for the SimSeq Bottomly data and $n_1=n_2=3$ for SimSeq mouse data) and $\alpha =0.05$ (lfdr adjustment). The plotted filtering methods and corresponding percentile percentages are given in the legend. Note that the range of the y-axis is chosen result-based; filtering methods with low power may not be visible on some plots

Figures 3 and 4 show the differences in power of the filters compared to no filtering as a function of $\pi _0$, *m*, or $n_1=n_2$ for multiplicity adjustment with lfdrs (Additional file 2: Fig. S16 for the BH procedure). The parameters $\pi _0$, *m*, or $n_1=n_2$ are either varying on the x-axis or are fixed at $\pi _0=0.8$, $m=10000$, $n_1=n_2=10$ (simulations for $\pi _0=0.99$ or based on other data sets, can be found in Additional file 2). The adaptive filter incorporates all thresholds from Table 2; however, the Figures only show the results for a selection of the considered thresholds: mean and max-based filters with the 1% and the 90% percentile, the zero-based filter with threshold $u=8$, and the Jaccard filter. In addition, the adaptive filter with filter parameter $l=5$ is plotted. Note that we use different scales on the y-axis to provide information on the methods with high power values in detail. Therefore, on some plots, filtering methods with very low power and large power differences are not visible. In most scenarios, this concerns the mean and max-based filter with the 90% percentile. Additional file 2: Figs. S3 and S16 show results for the NB simulation with distributed parameters according to the Bottomly data set, the second row for SimSeq simulation (Kidney data), and row 3 for the PROPER simulation. In Fig. 4, further NB sim distributed, SimSeq and PROPER simulations with varying sequencing depth as a function of $\pi _0$ are shown.

It can be seen that the improvement in power for the adaptive filter is only moderate compared to some of the individual filtering methods with a large number of rejections. However, the adaptive filter gives a larger power in all scenarios while individual filters perform well in tailored settings only. For instance, the Jaccard filter shows a good performance in a lot of scenarios, while for PROPER simulations with sequencing depth = 5*m* and $\pi _0=0.5$ (lfdr adjustment), the gain in power for the adaptive filter is up to 22 percentage points compared to the Jaccard filter, for $\pi _0=0.99$ the gain is only 0.7 percentage points. For the SimSeq Bottomly simulations, it is up to approximately 9 percentage points.

We investigated the FDR of the adaptive filter in the above simulation scenarios. In most scenarios with $m\ge 2500$ the simulated FDR was below the nominal level 0.05, if the filter parameter *l* is 5 or larger. This holds also under the global null hypothesis (see Additional file 2: Figs. S8–S11, S20, S21). For some simulation scenarios based on SimSeq several of the individual (non-adaptive) filter methods showed an inflated FDR (see Additional file 2: Fig. S7). For these simulations, especially filters that select only a small percentage of genes for testing inflate the FDR. As a consequence, also the FDRs of the adaptive filter are increased (see Additional file 2: Figs. S9 and S19): For lfdr adjustment, the observed maximum FDR for the SimSeq simulation based on the Bottomly data is 0.098 ($\pi _0=1$), for the mouse data 0.17 ($\pi _0=1$). For the latter, the FDR of $\pi _0=0.95$ and 0.99 is also increased to 0.1 and 0.16. For the PROPER simulations and the BH procedure the simulated FDR of the adaptive filtering ($l=5$) is inflated for $\pi _0=1$ and 0.99 to a maximum of 0.069 and 0.071 for large values of $m \ge 1000$ (Additional file 2: Fig. S21), no increase is observed for the lfdr method (Additional file 2: Fig. S11). However, as above, the increase in FDR for these scenarios is already observed for the individual (non-adaptive) filtering methods (see [18] for a detailed discussion of FDR control for count data). This increase causes also the inflation of the FDR of the adaptive filter.

In Additional file 2: Figs. S11 and S21 we also investigate the influence of smaller $m=\{100, 500\}$ on the FDR. For the lfdr method, for filter parameter $l=5$, the observed FDR is lower than $\alpha =0.05$ for all scenarios and all values of *m*. For the BH method, however, for $l=5$ and $m=100$ or $m=500$, the simulated FDR is larger than 0.05 in some scenarios with a maximum inflation of 0.062 for the PROPER simulation ($m=100$ and $\pi _0=0.8$). Small increases are also observed for NB distributed simulations and $m=100$ (maximum inflation 0.053). However, for these scenarios, for some of the individual filtering methods the observed FDR is larger than $\alpha$.

## Real data application

We applied the adaptive filtering method to several RNA-seq data sets from the literature. We used the Bottomly, mouse mammary, Kidney and Sultan data sets which we had already used in the data simulations. Additionally, we reanalysed the Airway dataset (library airway, [19]) and a random sample of this data set with halved sample sizes per group to generate an application with potentially lower power (Airway 2). Real data analyses were further conducted for a random sample of 10 samples per group from the Kidney data set (in order to have similar group sizes as in the simulation study) and the Yuen data set from an experiment with de novo assembled transcriptome as a reference for gene counting (see Table 3, additional information on the data sets can be found in Additional file 1). R source code can be found in Additional file 4.

**Table 3:** Description of data sets

Data set	m(% of genes with only zero counts)	$n_1/n_2$	Description
Kidney	20531 (3)	72/ 72	non-tumour versus tumour samples [6]
Kidney 2	20531 (5)	10/10	random sample of Kidney data set
Bottomly	36536 (35)	10/11	C57BL/6J versus DBA/2J (mice strains) [7]
Mouse mammary	27179 (21)	6/6	basal versus luminal cell types in mice [10]
Sultan	52580 (83)	2/2	human embryonic kidney versus B cell lines [8]
Airway	64102 (52)	4/4	Airway smooth muscle cell lines [19]
Airway 2	64102 (52)	2/2	random sample of Airway data set [19]
De novo assembly:		$n_1/../n_4$	
Yuen	96831 (12)	3/3/3/3	transcriptomes of lucinid clam of 4 organs [20]
Only data simulation:		$n_1$	
Cheung	52580 (76)$^1$	41	lymphoblastoid cell lines from unrelated
			individuals [11]

$^1$ only a subset of 17580 genes with a reduced percentage of genes with only zeros is used for data simulation

In the reanalyses first all genes with only zero counts were removed (basic filter). Second, the filtering strategies as described in Table 2 were applied. The number of rejections was calculated ($\alpha =0.05$) as well as the proportion of the filtered genes. Again, filtering was performed after data analysis (see order (a) in Methods section), in addition, we performed a second data analysis where filtering was performed before normalisation and data analysis (order (b), Additional file 2: Table S1).

Table 4 shows the resulting maximum number of rejections for the filtering method and the corresponding observed proportion of filtered genes (for the basic filter based on all *m* genes, for other filters based on the non-zero genes) for multiplicity adjustment with lfdr (in Additional file 2: Fig. S12 shows histograms of estimated lfdrs and Table S2 shows results for the BH procedure). The adaptive filter with the highest number of rejections is highlighted in bold. It can be seen that, in most cases, the max-based filter leads to the largest number of rejections, however, with differing proportions (between 4 and 80 for lfdr adjustment and 2 and 70 for BH procedure). In at least one example, the mean-based, zero-based and Jaccard filters also lead to the highest number of rejections. The ordering of the data pre-processing (Additional file 2: Table S1) has quite a large impact on the number of rejections. Whether it is better to filter at the very beginning or at the end depends on the data set and on the filtering method.

**Table 4:** Real data application

	No filter	Basic	Mean-based	Max-based	Zero-based	Jaccard
Bottomly	1443	1324 (35)	1488 (24)	**1489 (25)**	1417 (15)	1371 (34)
Sultan	-	2801 (83)	**3511 (11)**	3445 (15)	**3511 14)**	2864 (42)
Airway	0	1029 (48)	1554 (60)	1576 (60)	1235 (26)	**1612 (57)**
Airway 2	-	102 (52)	275 (80)	**347 (80)**	120 (16)	197 (54)
Mouse	9151	8772 (21)	9173 (6)	**9540 (18)**	9192 (5)	8593 (28)
Kidney	13076	13075 (3)	13282 (5)	**13299 (4)**	11784 (19)	13072 (2)
Kidney 2	5777	**6357 (5)**	**6357 (2)**	6355 (3)	**6357 (4)**	6355 (3)
Yuen						
gill vs. mantle	7932	7932 (6)	9912 (38)	**10340 (48)**	8619 (25)	7932 (0)
gill vs. foot	7534	7534 (8)	9537 (38)	**9685 (32)**	8194 (27)	7543 (0)
gill vs. vmass	6093	6093 (4)	8079 (44)	**8948 (55)**	7023 (23)	6093 (0)
mantle vs. foot	5291	5291 (12)	5802 (41)	**5911 (36)**	5470 (13)	5291 (0)
mantle vs. vmass	2468	2468 (6)	3655 (47)	**4007 (68)**	3034 (23)	2468 (0)
foot vs. vmass	3605	3605 (7)	5054 (35)	**5602 (66)**	4178 (27)	3605 (0)

Maximum number of rejections for each filtering method and the corresponding observed proportion of filtered genes in parentheses (for the basic filter based on all genes, for other filters on the non-zero genes) for several data sets, multiplicity adjustment with lfdrs, $\alpha =0.05$ Filtering is performed at the end (order (a)). The adaptive filter with the highest number of rejections is highlighted in bold

## Discussion

In this manuscript we investigate an adaptive filtering procedure where several filtering methods are considered and the filter leading to the largest number of rejections is chosen. The proposed strategy may mimic the actual practise. The more important it is to investigate the impact of such a procedure on the Type I error rate and power, as it is not self-evident that such a strategy is sound and does not lead to biased hypothesis tests. For example, it has been shown that in multiple testing problems where the familywise error rate is controlled, an adaptive approach without strict rules may generate a high inflation of the error rate (e.g., [21, 22]). In this manuscript we thus give some justification for the proposed strategy when a large number of hypotheses is tested controlling the FDR and investigate by simulations under which conditions (e.g, with regard to the number of hypotheses tested) FDR control holds.

It can be seen that the improvement in power for the adaptive filter is only moderate compared to some of the individual filtering methods with a large number of rejections. However, which filtering methods and thresholds lead to large power values is in practice unknown and compared to any particular filtering method the increase in power by the adaptive filter can be very large. The adaptive filtering method selects the best filtering method (in terms of rejections) without the need to pre-specify a single individual filtering method and therefore avoids the choice of an inappropriate filter leading to a large loss in power. We suggest to consider each filtering method with several thresholds covering a broad range in the adaptive filter. For example, in the simulation study we included the thresholds given in Table 2.

In this manuscript, we chose the maximum number of rejections *R* as the criterion for the adaptive filter. This criterion may be replaced by, e.g., a post-hoc power estimator [23]. Here, after data analysis for each filter the post-hoc power defined as the proportion of truly rejected null hypotheses is estimated. The filtering method with the highest post-hoc power is then chosen as the adaptive filter.

To adjust for multiple testing we consider two different approaches, the lfdr and the BH method. Our results reveal that for both methods the adaptive filtering strategy is well applicable. The BH method might have the advantage of higher power values but less robustness, e.g., when genes are correlated and the p-value distribution does not follow a uniform distribution under the null hypothesis. Thus we do not recommend a specific method for multiplicity control but a researcher has to decide individually based on the experiment which method should be used. The BH and lfdr method are in fact two different concepts to control for multiplicity when several simultaneous hypothesis tests are performed. Control of the FDR at significance level $\alpha$ essentially means that the expected value of the proportion of false rejections under all rejections is equal to or smaller than $\alpha$. Control of the FDR is an overall characteristic of the multiple testing procedure, which does not distinguish between rejected hypotheses. In contrast, lfdr is computed for each hypothesis and can be interpreted as the posterior probability that the null hypothesis is true, conditional on the observed test statistic or p-value. It has been shown (e.g., [14] or [24]), that these concepts are related, as the FDR essentially corresponds to an average of the lfdrs of rejected hypotheses. Therefore when controlling the lfdr at level $\alpha$, the FDR is controlled at a level smaller than $\alpha$. See also the discussions on the concepts and methods to control the lfdr and FDR in, e.g., [25–27].

For the analysis of RNA-seq data, several approaches have been proposed [28], e.g, based on the Poisson or the NB distribution, but no “optimal” method has been defined. Popular methods include, e.g., limma/voom [29], EdgeR [4], Deseq2 [30], or the non-parametric SAMseq method [31]. Adaptive filtering is equally applicable for all of these methods. It has, however, been shown [18, 32], that scenarios exist, where some of the proposed methods might not control the FDR. For our simulations we applied the limma/voom method, as, e.g., suggested by [18]. The results in Additional file 2: Figs. S8–S11, S20, S21 show that in most scenarios the FDR of the adaptive filter is maintained at level $\alpha$ for $l=5$ or even $l=0$. Still, there exist some scenarios where the FDR is increased. However, this is not due to the adaptive filtering but due to fact that some of the individual (non-adaptive) filters do not control the FDR. In these cases the limma/voom procedure or the method for multiplicity adjustment (lfdr or BH) might not be appropriate. If the FDR is increased for one or several individual filters, it consequently might be increased when many filters are considered for the adaptive filter.

The properties of the filtering methods depend on the distribution of the collected data and thus on the applied technology. Here, we focus on RNA-seq data; however, the proposed adaptive filter approach can also be applied for other types of high-dimensional data, such as, e.g. for microarray data.

## Conclusions

In RNA-seq studies, filtering is an important processing step; however, there is no consensus on the choice of the filtering process or the threshold values. We investigated different simulation strategies and showed that it is not possible to identify a filter which is optimal for all simulation scenarios. Depending on the distributional scenario, mean or max-based filters with adequate thresholds, the Jaccard, or zero-based filters maximise the number of rejections. In many scenarios, the basic filter alone may be a good choice. This holds for many NB simulations with distributed parameters and is in line with the findings of Rau et al. (2013) [2]. However, for simulations, where the dependence structure between genes and distribution of dispersion and mean parameter is based on real data, and the real data analyses, a distinct improvement in the number of rejected hypotheses is observed if more advanced filtering methods are applied.

The proposed adaptive filtering procedure has practically no impact on the FDR in many simulation scenarios, if the filter leading to the largest number of rejections is selected and no additional adjustment for multiplicity for the number of considered filters is performed. If, however, the proposed adaptive procedure rejects only a small number of hypotheses, the researcher has to stick to a previously defined reference filter to avoid FDR inflation. For small number of hypotheses *m* we have no theoretical proof that FDR control of the procedure can be guaranteed. We show by simulations, that for finite *l*, FDR control can be achieved by choosing a larger threshold *l* if *m* is small. Exceptions were observed for some scenarios of the SimSeq, PROPER and NB distributed simulations. However, for these, some of the (non-adaptive) filters do not control the FDR, and thus also the adaptive filter inflates the level (as explained in the Discussion). Note that choosing a larger threshold *l* comes at the cost that the procedure becomes less adaptive, because only filters can be chosen, where more than *l* hypotheses are rejected. While we cannot provide analytical formulas to guide the choice of *l*, we show by simulations that, in the considered scenarios, for $m \ge 1000$ and $l=5$, no inflation of the FDR by the adaptive filter is observed, even under the global null hypothesis. For $l=3$ FDR control is observed in these scenarios when $m\ge 2500$.

## Methods

We consider an RNA-seq experiment comparing two groups of *n* independent samples with *m* null hypotheses $H_{0i}$, $i=1,\dots ,m$. The two groups are of size $n_1$ and $n_2$ with $n_1+n_2=n$. The read count for gene *i* in sample *j*, $j=1,\dots ,n$, and group *g*, $g\in \{1,2\}$, is denoted by $C_{ijg}$. We focus on two-sided hypotheses $H_{0i}: \mu _{1i}=\mu _{2i}$ versus $H_{1i}: \mu _{1i}\ne \mu _{2i}$, where $\mu _{1i}$ and $\mu _{2i}$ denote the expected counts in groups 1 and 2 of the *i*-th gene. For all simulated data sets, the trimmed mean of M-values normalisation method (TMM) was applied where reads are scaled by weighted log-fold-change values of a reference sample [13]. For the data analysis, we transformed the count data via the voom function and applied a linear model for each gene with the limma package in Bioconductor [29, 33]. The voom function converts the discrete read counts to continuous log2-counts per million and the estimated variances are used as weights for weighted least square regression. To adjust for multiple testing, we show results for two different strategies: For one part of the simulations, the lfdr is estimated for each gene and all hypotheses with lfdr lower than the significance threshold $\alpha =0.05$ are rejected. The lfdr for hypothesis *i* is defined as the posterior probability in a Bayesian mixture model that for hypothesis *i* the null hypothesis holds (for detailed explanations, see [15, 34]). For the calculation of the lfdrs the R-package fdrtool [35] was applied to the vector of p-values with cutoff method false non-discovery rate and using a modified Grenander approach for density estimation [15]. Second, we performed simulations where the BH procedure was applied to adjust for multiplicity and to control the FDR of the experiment at level $\alpha =0.05$.

The order of data processing (filtering, normalisation and data analysis) is not definite. Normalisation is performed before the data analysis, yet, there are different approaches on when to apply the filtering step in the analysis pipeline [2, 28]. In the simulations and real data applications, we apply the following analysis pipelines: (a) normalise the data, perform the analysis and then perform the filtering step using the raw or the normalised data to reduce the set of genes. The multiple testing procedure is performed with the reduced set of genes after filtering; (b) first filter the raw data, then normalise them and perform the analysis.

### Adaptive filter

We propose a new filtering strategy which combines several filtering methods:

1. First, a set of *F* different filters, $F>0$, is defined, where one of the filtering methods is specified as reference filter. An integer filter parameter *l* is specified.
2. For a given data set, the *F* filters are applied. Each filter generates a set of candidate genes of different magnitude and for each set the data analysis is performed (or has been performed before, if pre-processing order (a) has been chosen). Then, multiple testing procedures are applied for each of the *F* sets of p-values and the resulting numbers of rejected hypotheses $R_1,...,R_F$ are calculated. $R_f$, $f=1,\dots ,F$, denotes the number of rejected hypotheses for filtering method *f*.
3. To determine the definite final analysis, the filtering method *k* is chosen where the largest number of hypotheses are rejected,
  $$\begin{aligned} k=\text {arg max}_{f=1,\dots , F} R_f. \end{aligned}$$
  However, if $R_k<l$, only the results of the reference filter may be applied.

The adaptive filter chooses the filtering strategy with the largest number of rejections; however, the multiple testing procedure - in our case lfdr or BH procedure, adjusts only for the number of genes but not for the number of filtering strategies. Thus, in principle, the FDR may be increased when the filtering method leading to the largest number of rejections is selected. However, we showed in the simulations, that the observed FDR applying the adaptive filter is below $\alpha$ in many scenarios, as long as the FDR for each filter individually is below $\alpha$. Hereafter we give a heuristic, asymptotic argument based on earlier work on sequential multiple tests controlling the FDR [36, 37], focusing on the BH procedure (which is more liberal than the lfdr controlling procedure). We assume that for each filtering method the FDR is controlled and consider two scenarios. First, assume that for a positive fraction of hypotheses the alternative holds ($\pi _0<1$). Then, if for each filter the selected test statistics are sufficiently independent and additional technical conditions apply [36], the BH procedure asymptotically controls the false discovery proportion (FDP), defined as the fraction of erroneously rejected hypotheses among all rejected hypotheses, and not only the FDR, its expected value (see, e.g., [38]). Thus, as the number of hypotheses increases, the FDPs for each filtering method are bounded almost surely by $\alpha$. Consequently, this also holds for the maximum FDP across all considered filters. Therefore, the level of the multiple test using the adaptive filter is asymptotically bounded by $\alpha$ (compare Theorem 1 in [36]). In the second scenario, under the global null hypothesis ($\pi _0=1$), the BH procedure does not control the FDP (as it is either 0 or 1 in this case) and the above argument cannot be applied. However, in this case asymptotic FDR control follows because the adaptive filter chooses the reference filter if none of the filters leads to more than *l* rejections: Choosing $l_m=q m,$ for some $q>0$ and assuming that the filtered p-values are independent, it follows, that the probability that more than $l_m$ hypotheses are rejected by the BH procedure, converges to 0. In this case the adaptive filter selects almost surely the reference filter. As the multiple test based on the reference filter controls the FDR, this follows also for the adaptive filter (see Theorem 2 in [36]).

It has been shown [18] that for some analysis methods as, e.g., limma/voom, EdgeR, or Deseq2, scenarios exist, where after adjustment for multiplicity the actual FDR level is not maintained at the predefined level $\alpha$ for each filter individually. In this case, also the adaptive filter might not control the FDR at level $\alpha$.

## Supplementary Information

**Additional file 1.** Additional information on simulation strategies and on real data is presented.

**Additional file 2.** Extension of the simulation studies from the manuscript (additional data sets, parameters and modified order of data processing, BH procedure, values of simulated FDRs and influence of filter parameter l; distribution of lfdrs in real data).

**Additional file 3.** Data example for the R-code from Additional file 4.

**Additional file 4.** Example code for real data example for programming language R.

## Data Availability

For the simulation studies and the real data application the following data sets were used (all of them publicly available, only the Yuen data were provided by the authors): Kidney [6]: Data obtained from R-package SimSeq [9]. Bottomly [7]: Available at http://bowtie-bio.sourceforge.net/recount/. We obtained the data from the R-package dexus [39]. Mouse mammary [10]: Available at https://figshare.com/s/1d788fd384d33e913a2a. Sultan [8]: Available at http://bowtie-bio.sourceforge.net/recount/. Airway: Data obtained from R-package airway [19]. Yuen [20]: Raw data provided by the authors. Cheung [12]: Data obtained from R-package PROPER [11].

## Abbreviations

BH: Benjamini–Hocberg
CPM: counts per million
DE: differentially expressed
FDP: False discovery proportion
FDR: False discovery rate
lfdr: Local false discovery rate
NB: Negative binomial
RNA-seq: RNA sequencing
TMM: trimmed mean of M-values normalization
