# Supplementary material for: Impact of adaptive filtering on power and false discovery rate in RNA-seq experiments
Source: BMC Bioinformatics. 2022 Sep 24;23:388. doi: 10.1186/s12859-022-04928-z (PMC9509565; doi:10.1186/s12859-022-04928-z)
Supplement: Supplementary file 1 — Additional file 1. Additional information on simulation strategies and on real data is presented. [file 12859_2022_4928_MOESM1_ESM.pdf]

Impact of adaptive filtering on power and False Discovery Rate in RNA-seq experiments
Additional file 1

Sonja Zehetmayer, Martin Posch, Alexandra Graf

January 25, 2022

Details on simulation scenarios

In the following we give details on the simulation scenarios as described in the manuscript in Table 1. Different simulation strategies are investigated under different scenarios to show the influence of filtering and the performance of the adaptive filter on power.

1 NB simulation

The NB simulation is based on the very simplistic assumption that each gene follows the same negative binomial distribution (NB) with constant dispersion parameter $r = 5$ and constant mean parameter $\mu = 5$ for the π_0 null hypotheses and $\mu = 15$ for the $1 - \pi_0$ alternative hypotheses (for half of the alternative hypotheses, the higher effect was allocated to group 1 and for the other to group 2).

2 NB simulation with distributed dispersion and mean values

This second type of simulation scenario extends the NB simulation: The dispersion parameter r and the mean parameter μ of the negative binomial distribution (NB) are vectors with individual values for each gene. These vectors are chosen based on real RNA-seq data sets. This simulation scenario was performed according to Rau *et al.* (2013). Three real data sets consisting of several replicates for two groups were used: Bottomly *et al.* (2011), Sultan *et al.* (2008) (both as in Rau *et al.*, 2013), and Kidney data (The Cancer Genome Atlas Research Network, 2013). A description of the real data sets can be found in Table 3 in the manuscript.

To obtain parameters for the simulation study, the following steps were performed:

- For each data set, genes containing only zeros in one of the two groups and mean count < 5 were removed.
- The DESeq Bioconductor package (Anders and Huber, 2010) was applied to analyse the data based on a model using the negative binomial distribution and to identify differentially expressed genes for the simulation study (all genes with adjusted

p-values according to the Benjamini-Hochberg method smaller than 0.05). In addition DESeq analysis was used to fit the relation between dispersion estimates r_i per hypothesis i, $i = 1,\dots,m$, and corresponding means μ_i over both samples (via gamma regression) for each data set with the following results:

$$\text{Bottomly: } r_i = 0.03 + 0.88/\mu_i \quad i = 1\dots,m \tag{1}$$

$$\text{Kidney: } r_i = 0.27 + 61.61/\mu_i \quad i = 1,\dots,m \tag{2}$$

$$\text{Sultan: } r_i = 0.07 + 1.2/\mu_i \quad i = 1,\dots,m \tag{3}$$

- The mean parameters for the simulation study were then fixed as follows: Mean parameters for hypotheses which are not defined differentially expressed by the DESeq procedure were set to the overall mean of the corresponding hypothesis for both groups. If a hypothesis was defined as differentially expressed, the mean parameter for group 1 (group 2) was set to the empirical mean of group 1 (group 2).
- The dispersion parameters were calculated according to Equation (1)-(3) as a function of the overall mean μ_i over both samples for each hypothesis. Additionally, dispersion parameters for hypotheses with overall mean < 20 were set to 10^{-10}

3 SimSeq Simulation

The SimSeq simulation is a data-based simulation algorithm without parametric assumption on the distribution, which tries to match the dependence structure of RNA-seq data (R-package SimSeq, Benidt and Nettleton (2015)). Data columns are subsampled from real data and individual read counts are randomly selected adjusted by a correction factor to generate differential expression. Thus no assumption on the distribution of the read counts is made. First for each gene from the real data set Wilcoxon Rank sum tests and corresponding p-values are calculated. Local false discovery rates are then calculated to derive weights. Depending on this weights, genes are assumed to belong to the null hypothesis or to the alternative hypothesis. The randomly selected genes are adjusted by some normalizing factors estimated from the real data (a positive numeric vector of multiplicative normalization factors for each column of the counts matrix. As proposed in the SimSeq help we apply the 75% percentile of each sample column.) and by a correction factor to generate differential expression. Real data sets Bottomly (Bottomly *et al.* (2011)), Kidney (The Cancer Genome Atlas Research Network (2013)), and mouse (Fu *et al.* (2015)) were applied. Note that the real data must be sufficiently large to get datasets that are sufficiently different from each other.

4 PROPER Simulation

The following simulation procedure is based on the bioconductor package PROPER (Wu *et al.*, 2015): Read counts are assumed to follow an NB distribution for gene-wise individual mean and dispersion parameters, adjusted by some normalizing factor (e.,g. library size). First a baseline expression level for each gene is resampled non-parametrically using empirical average expression estimated from an existing data set. In our simulation study we apply the Cheung data (Cheung *et al.*, 2010)). Then the dispersion parameter is again resampled based on empirical sample dispersions. For the log fold change for

differentially expressed genes, random values are sampled from a normal distribution, in our simulations from $N(0,2)$.

In the simulation study either baseline expressions or sequencing depth can be specified. Sequencing depth is the total number of reads for each sample and it is assumed that all experiments have the same total sequencing depth. Thus in the simulations the mean parameters are adjusted to get the same sequencing depths. We performed several simulation studies with sequencing depths 50000, 100 000 and 500 000 and compare the impact of the adaptive filter on sequencing depth.

Note that in the Cheung data set many of the baseline expressions are zero as they are non-expressed. If, by chance, a gene with zero baseline expression is sampled, the simulation study generates a gene with only zero reads, regardless of belonging to the group of differentially expressed or non-expressed genes. The same happens if a fixed sequencing depth was chosen. Thus additional noise is generated by this proceeding. In our simulation study we limited the number of baseline expressions of value zero to obtain useful power values.

Details on real data sets

Kidney: Kidney renal clear cell carcinoma RNA-seq dataset (The Cancer Genome Atlas Research Network, 2013) from The Cancer Genome Atlas project. The samples stem from a heterogeneous population differing in race, gender, age and ethnicity. The data were sequenced using the Illumina HiSeq 2000 RNA Sequencing Version 2 analysis platform and are available from the SimSeq package (Benidt and Nettleton, 2015). The data set includes 20531 genes from 72 pairs of matched columns with two samples from each individual with Kidney renal clear cell carcinoma. One sample comes from a tumorous region and one from a non-tumourous region of the body.

Bottomly: Homogeneous samples with genetically identically mice (Bottomly *et al.*, 2011). The data set includes 21 striatum samples from two stains of inbreed mice (C57BL/6J and DBA/2J). Since the data are from inbreed animal models, the biological variations among replicates are small. The counts were produced using an Illumina GAIIx sequencing machine. We obtained the data from the R-package dexus (Klambauer *et al.*, 2013).

Cheung: Expressions of lymphoblastoid cell lines from 41 individuals from the International HapMap Project (Illumina technology). The data are from unrelated individuals (grandparents), so the expressions show large biological variations overall (Cheung *et al.*, 2010). Data were obtained from bioconductor package PROPER (Wu *et al.*, 2015).

Mouse mammary: Basal versus luminal cell types in mice (Fu *et al.*, 2015), where cell survival versus cell death in mammary was explored after deleting the pro-survival gene Mcl-1 in the mammary epithelium (Illumina HiSeq 2000).

Sultan: High-throughput sequence of the human transcriptome from a human embryonic kidney and a B cell line (Illumina deep sequencing). In each group 2 samples are investigated (Sultan *et al.*, 2008).

Airway: To understand the mechanism by which glucocorticoids suppresses inflammation in the airway smooth muscle, transcriptomic changes in four primary human airway smooth muscle cell lines that were treated with dexamethasone were investiated with RNA-seq (Illumina) (Himes *et al.*, 2014).

Yuen: Transcriptomes (Illumina) of lucinid clam (Loripes orbiculatus) from gills, foot, visceral mass (vmass), and mantle for differential expression analyses (Yuen *et al*, 2019). 12 RNA-Seq libraries were sequenced from three replicates each of four organs (de novo assembled transcriptome). For demonstrating purposes we make separate pairwise comparisons for each of the organs.

References

Anders,S., and Huber,W. (2010) Differential expression analysis for sequence count data *Genome Biology* **11: R106**, 1-12.

Benidt,S. and Nettleton,D. (2015) SimSeq: a nonparametric approach to simulation of RNA-sequence datasets *Bioinformatics* **31**, 2131-2140.

Bottomly,D., Walter,N.A.R., Hunter,J.E., Darakjian,P., Kawane,S., Buck,K.J., Searles,R.P., Mooney,M., McWeeney,S.K., Hitzemann, R. (2011) Evaluating gene expression in C57BL/GJ and DBA/2J mouse striatum using RNA-seq and microarrays *PLoS One*, **6:3**, e17820, 1-8.

Cheung,VG and Nayak,RR and Wang,IX and Elwyn,S and Cousins,SM and Morley,M and Spielman,RS (2010) Polymorphic Cis- and Trans-Regulation of Human Gene Expression *PLoS Biology*, **8:9**, 1-14.

Fu,NY and Rios,AC and Pal,B and Soetanto,R and Lun,AT and Liu,K and Beck,T and Best,SA and Vaillant, F and Bouillet,P and Strasser,A and Preiss,T and Smyth,G.K and Lindeman,GJ and Visvader,JE (2015) EGF-mediated induction of Mcl-1 at the switch to lactation is essential for alveolar cell survival *Nature Cell Biology* **17:4**, 365375.

Himes,EB and Jiang,X and Wagner,P and Hu,R and Wang,Q and Klanderman,B and Whitaker,MR and Duan,Q and Lasky-Su,J and Nikolos,C and Jester,W and Johnson,M and Panettieri,AR and Tantisira,GK and Weiss,TS and Lu,Q (2014) RNA-Seq Transcriptome Profiling Identifies CRISPLD2 as a Glucocorticoid Responsive Gene that Modulates Cytokine Function in Airway Smooth Muscle Cells *PLoS One* **9:6**, 113.

Klambauer,G., and Unterthiner,T., and Hochreiter, S. (2013) DEXUS: Identifying Differential Expression in RNA-Seq Studies with Unknown Conditions. *Nucleid Acids Research* **41(21)**, e198-e198.

R Core Team (2017). R: A language and environment for statistical computing. R Foundation for Statistical Computing, Vienna, Austria. https://www.R-project.org/.

Rau,A., Gallopin,M., Celeux,G., Jaffrzic,F. (2013) Data-based filtering for replicated high-throughput transcriptome sequencing experiments, *Bioinformatics*, **29**, 17, 21462152. doi:10.1093/bioinformatics/btt350.

Sultan,M., Schulz,M.H., Richard,H., Magen,A., Klingenhoff,A., Scherf,M., Seifert,M., Borodina,T., Soldatov,A., Parkhomchuk,D., Schmidt,D., O'Keeffe,S., Haas,S., Vingron,M., Lehrach,H., Yaspo,M.L. (2008) A global view of gene activity and alternative splicing by deep sequencing of the human transcriptome *Science*, **15**, 956960.

The Cancer Genome Atlas Research Network (2013) Comprehensive molecular characterization of clear cell renal cell carcinoma *Nature*, **499**, 43-49.

Wu,H and Wang,C and Wu,Z (2015) PROPER: comprehensive power evaluation for differential expression using RNA-seq *Bioinformatics*, **31:2**, 233-241.

Yuen,B., Polzin,J., and Petersen,J.M. (2019) Organ transcriptomes of the lucinid clam Loripes orbiculatus (Poli, 1791) provide insights into their specialised roles in the biology of a chemosymbiotic bivalve *BMC Genomics*, **20:820**
