# Supplementary material for: Impact of adaptive filtering on power and false discovery rate in RNA-seq experiments
Source: BMC Bioinformatics. 2022 Sep 24;23:388. doi: 10.1186/s12859-022-04928-z (PMC9509565; doi:10.1186/s12859-022-04928-z)
Supplement: Supplementary file 2 — Additional file 2. Extension of the simulation studies from the manuscript (additional data sets, parameters and modified order of data processing, BH procedure, values of simulated FDRs and influence of filter parameter l; distribution of lfdrs in real data). [file 12859_2022_4928_MOESM2_ESM.pdf]

Impact of adaptive filtering on power and False Discovery Rate in RNA-seq experiments
Additional file 2

Sonja Zehetmayer, Martin Posch, Alexandra Graf

April 12, 2022

Contents

1 Power comparison for lfdr adjustment **2**
1.1 Comparison of individual filter strategies 2
1.2 Comparison of individual filter strategies: Simulations based on additional real data sets 3
1.3 Adaptive filter for $\pi_0 = 0.99$ 4

2 Simulated FDR values for lfdr adjustment **5**
2.1 Comparison of individual filter strategies 5
2.2 Comparison of individual filter strategies: Simulations based on additional real data sets 8
2.3 Adaptive filter: FDR values for $l = 0$ and $l = 5$ 9
2.3.1 Influence of filter parameter l and number of hypotheses m on FDR values 12

3 Distribution of lfdr in real data sets **13**

4 Power comparison for Benjamini-Hochberg procedure **14**
4.1 Comparison of individual filter strategies 14
4.2 Adaptive filter 17

5 Simulated FDR values for BH adjustment **18**
5.1 Individual filter strategies 18
5.2 Adaptive filter: FDR values for $l = 0$ and $l = 5$ 21
5.2.1 Influence of filter parameter l and number of hypotheses m on FDR values 22

6 Comparison of individual filter strategies: Modified order of data processing **23**

7 Real data application **25**
7.1 Modified order of data preprocessing 25
7.2 BH procedure 26

1 Power comparison for lfdr adjustment

1.1 Comparison of individual filter strategies

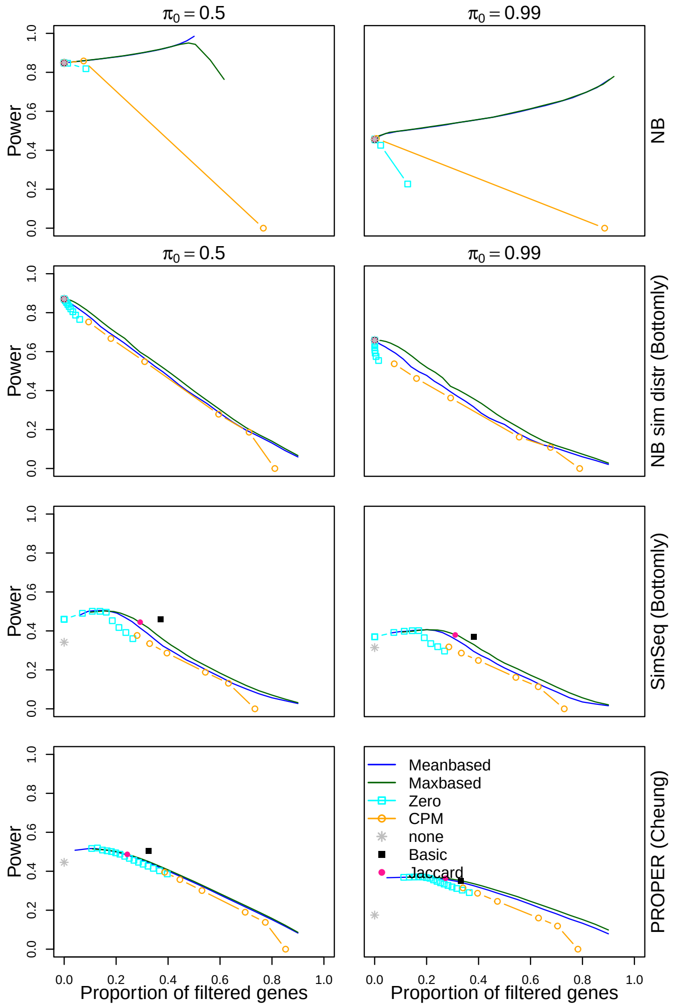

Figure 1: Power comparison of different filters, extension of Figure 1 in the manuscript for $\pi_0 = 0.5$ and 0.99 (lfdr adjustment): Power values for several filters and simulation strategies for $\alpha = 0.05$, $m = 10000$, $n_1 = n_2 = 10$ (or $n_1 = n_2 = 5$ for SimSeq (Bottomly)). The power of each filtering method is plotted as a function of the actual mean proportion of filtered genes across all simulation runs for the set of genes with at least one non-zero count, only the proportion of the basic filter is based on the total number of hypotheses m. The basic, Jaccard and no filter results are represented by a point because these methods are based on a fixed threshold. Note that: In the simulation preparation it turned out that the simulation based on the Bottomly data set for $\pi_0 < 0.8$ cannot be performed, as a too small number of alternatives for the simulations were identified. Thus for the Bottomly data simulation, $\pi_0 \in \{0.9, 0.99\}$.

1.2 Comparison of individual filter strategies: Simulations based on additional real data sets

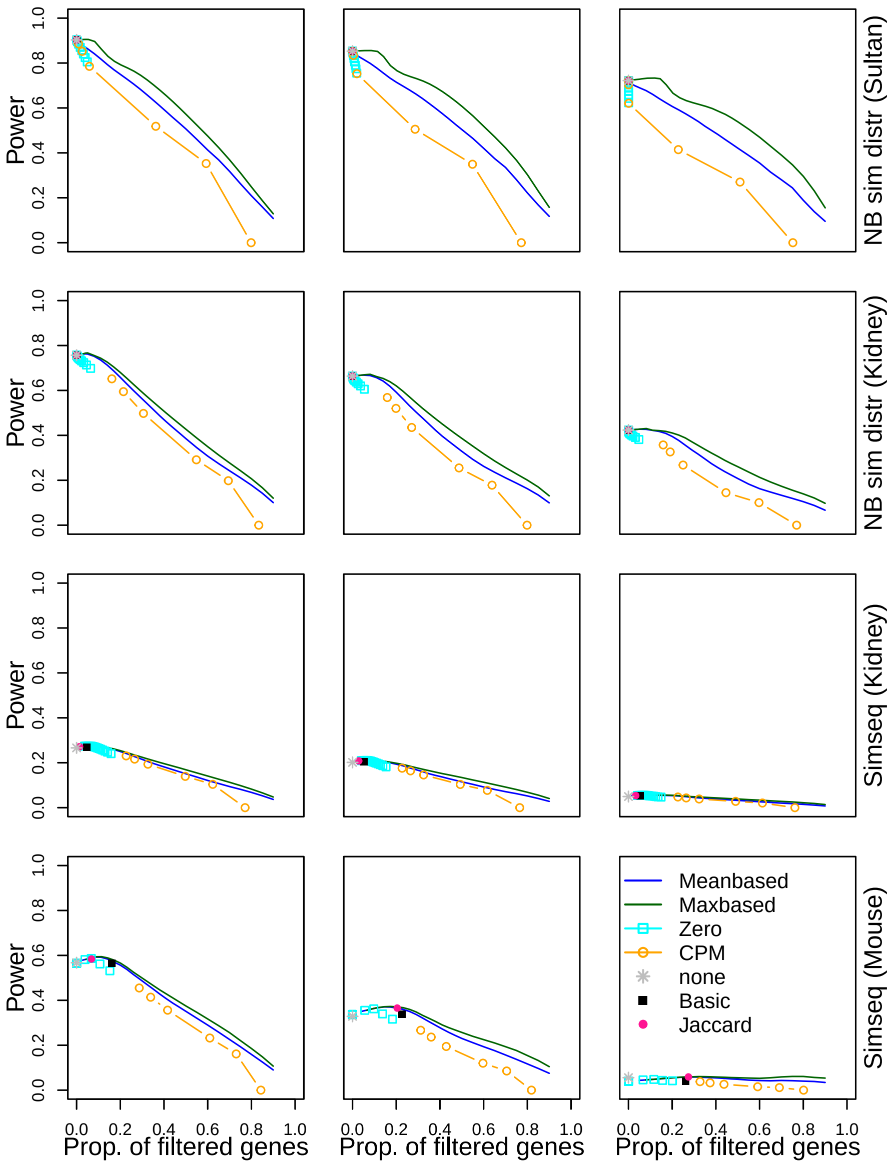

Figure 2: Power values and percentile thresholds for several filtering methods for $\pi_0 \in \{0.5, 0.8, 0.99\}$ (from left to right), $m = 10000$, $n_1 = n_2 = 10$, and $\alpha = 0.05$ for several simulation strategies. The power of each filtering method is plotted as a function of the actual mean proportion of filtered genes across all simulation runs for the set of genes with at least one non-zero count, only the proportion of the basic filter is based on the total number of hypotheses m. The basic, Jaccard and no filter results are represented by a point because these methods are based on a fixed threshold.

1.3 Adaptive filter for $\pi_0 = 0.99$

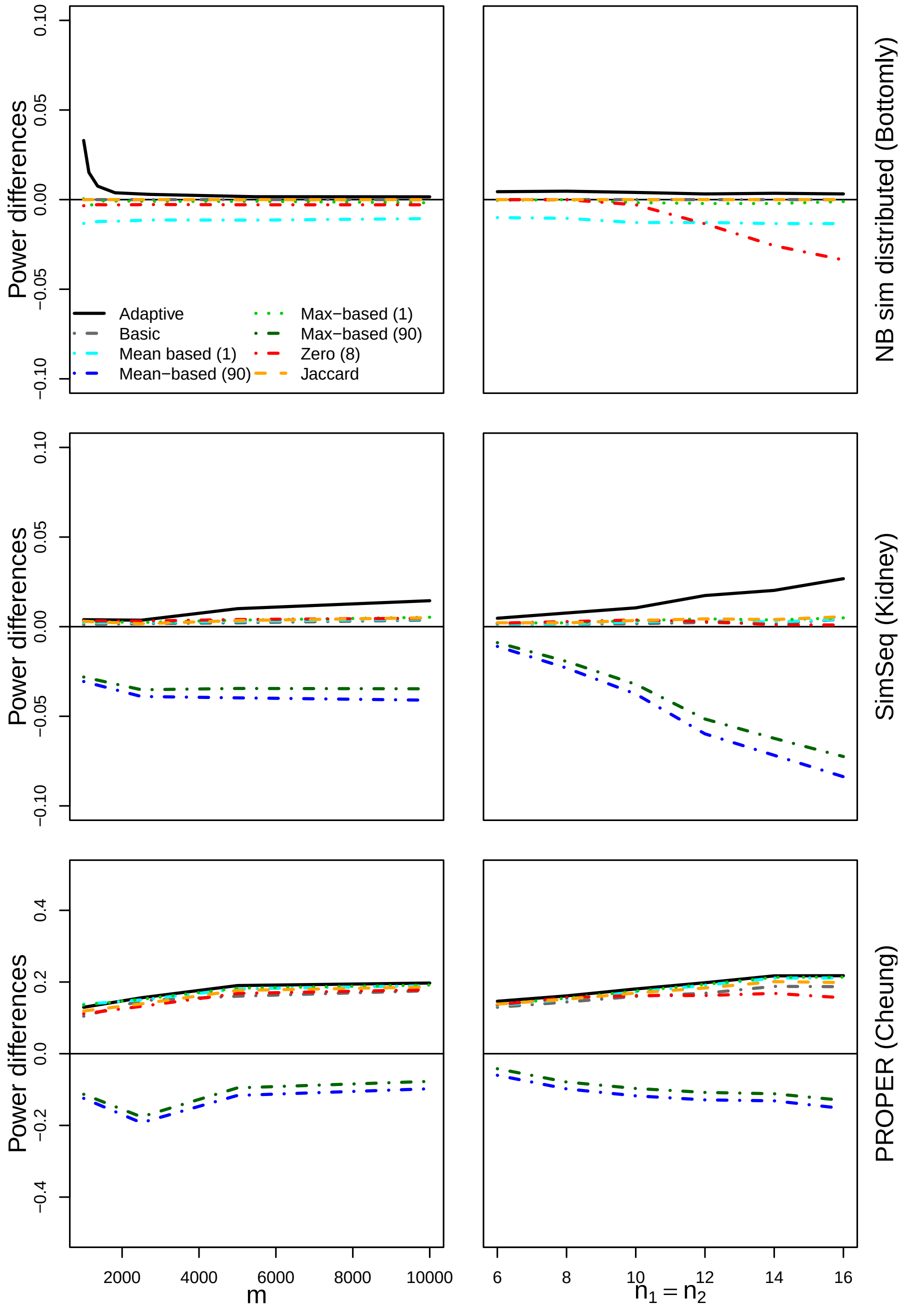

Figure 3: Differences in power for adaptive filter and selection of applied filter compared to no filter for NB sim distributed (Bottomly data set), SimSeq simulation (Kidney data set) and PROPER simulation (Cheung data) for varying m, or $n_1 = n_2$, respectively (lfdr adjustment). The plotted filtering methods and the corresponding percentile percentages are given in the legend. If not a parameter on the x-axis, $\pi_0 = 0.99$, $m = 10000$, $n_1 = n_2 = 10$, and $\alpha = 0.05$. Note that the range of the y-axis is chosen result-based, on some plots, filtering methods with low power may not be visible.

2 Simulated FDR values for lfdr adjustment

2.1 Comparison of individual filter strategies

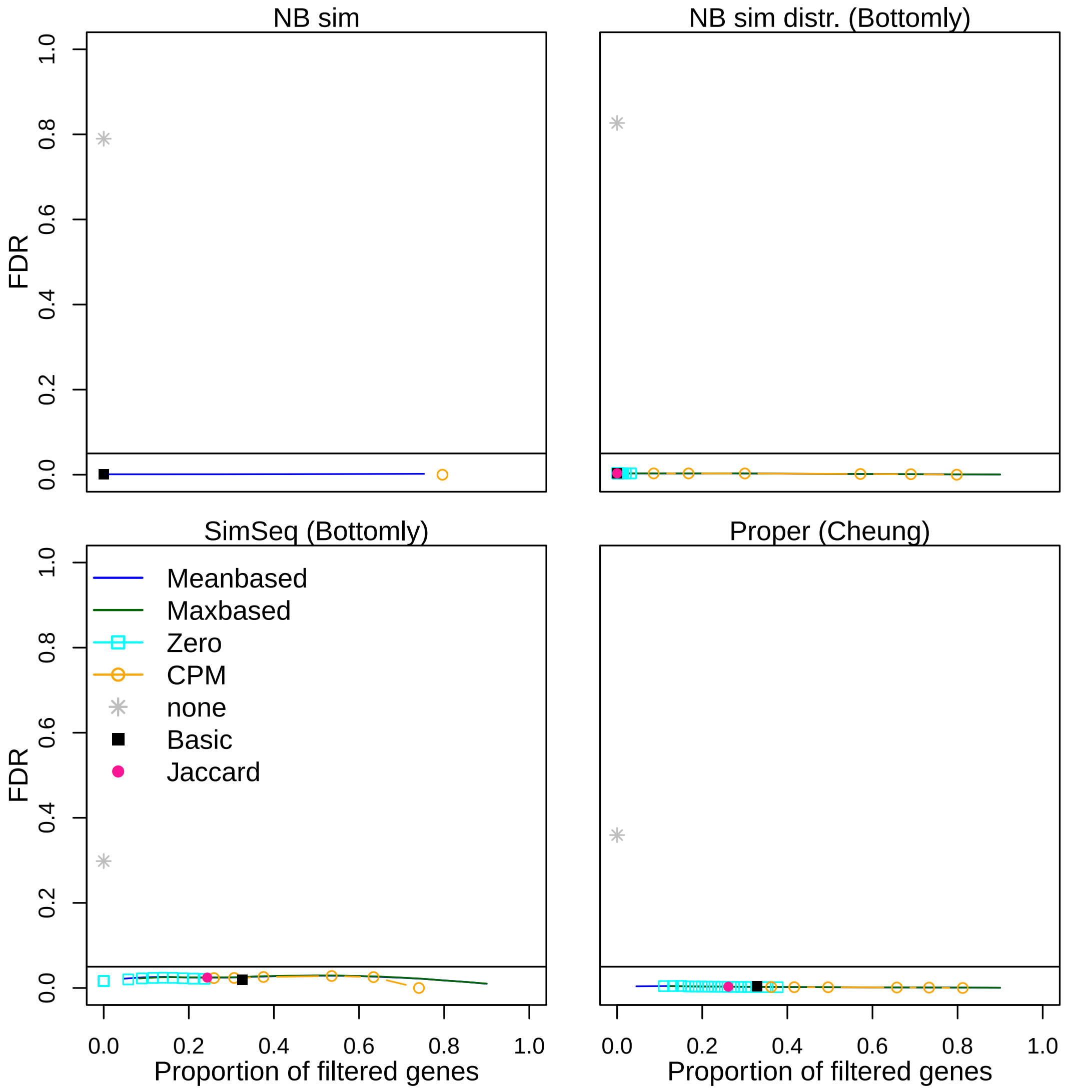

Figure 4: Simulated FDRs of different filters for results of Figure 1 from the manuscript (lfdr adjustment). FDR values for several filtering methods and simulation strategies for $\alpha = 0.05$, $\pi_0 = 0.8$, $m = 10000$, $n_1 = n_2 = 10$ (or $n_1 = n_2 = 5$ for SimSeq (Bottomly)). The FDR of each filtering method is plotted as a function of the actual mean proportion of filtered genes across all simulation runs for the set of genes with at least one non-zero count; only the proportion of the basic filter is based on the total number of hypotheses m. The basic, Jaccard and no filter results are represented by a point because these methods are based on a fixed threshold.

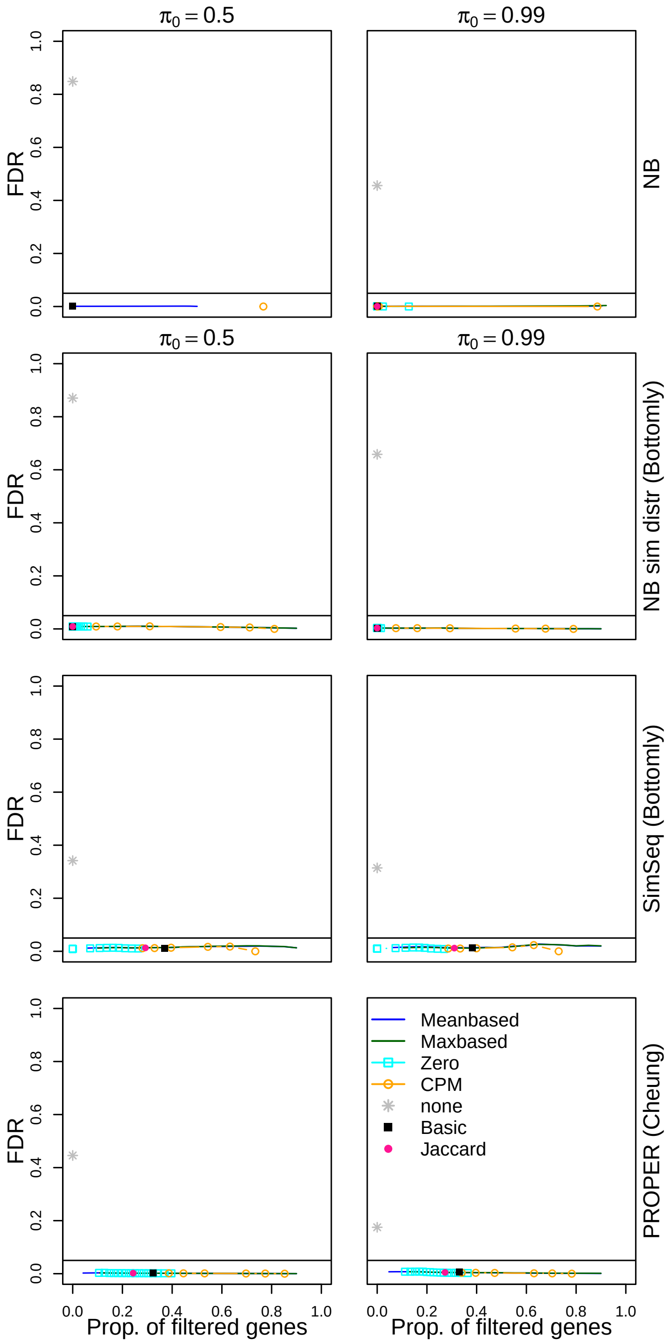

Figure 5: Simulated FDRs of different filters for result of Figure 1 from additional file 2: FDRs for several filters and simulation strategies for $\alpha = 0.05$, $m = 10000$, $n_1 = n_2 = 10$ (or $n_1 = n_2 = 5$ for SimSeq (Bottomly)) (lfdr adjustment). The FDR of each filtering method is plotted as a function of the actual mean proportion of filtered genes across all simulation runs for the set of genes with at least one non-zero count, only the proportion of the basic filter is based on the total number of hypotheses m. The basic, Jaccard and no filter results are represented by a point because these methods are based on a fixed threshold. For the Bottomly data simulation, $\pi_0 \in \{0.9, 0.99\}$.

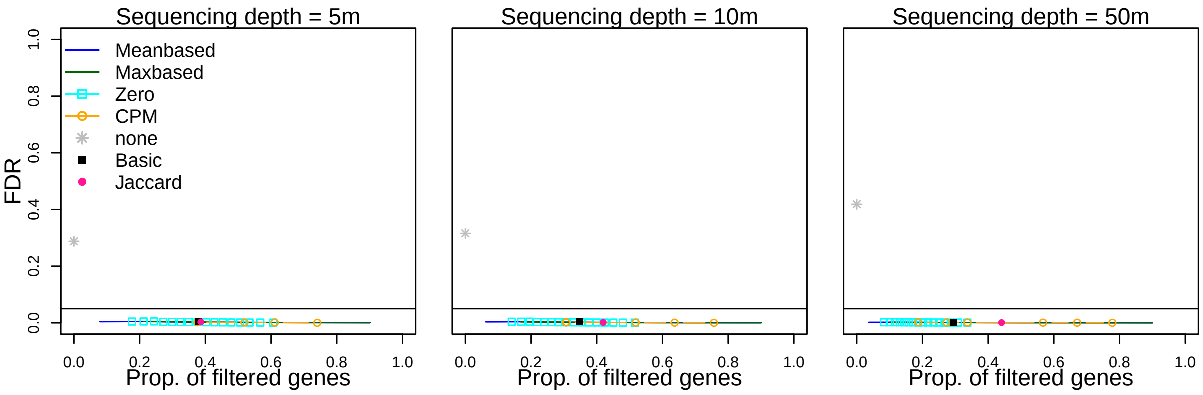

Figure 6: Simulated FDRs of different filters and sequencing depths for results of Figure 2 from the manuscript. FDR values for several filtering methods for PROPER simulation for $\alpha = 0.05$, $\pi_0 = 0.8$, $m = 10000$, $n_1 = n_2 = 10$ and sequencing depths $5m$, $10m$, and $50m$, respectively. The FDR of each filtering method is plotted as a function of the actual mean proportion of filtered genes across all simulation runs for the set of genes with at least one non-zero count; only the proportion of the basic filter is based on the total number of hypotheses m. The basic, Jaccard and no filter results are represented by a point because these methods are based on a fixed threshold.

2.2 Comparison of individual filter strategies: Simulations based on additional real data sets

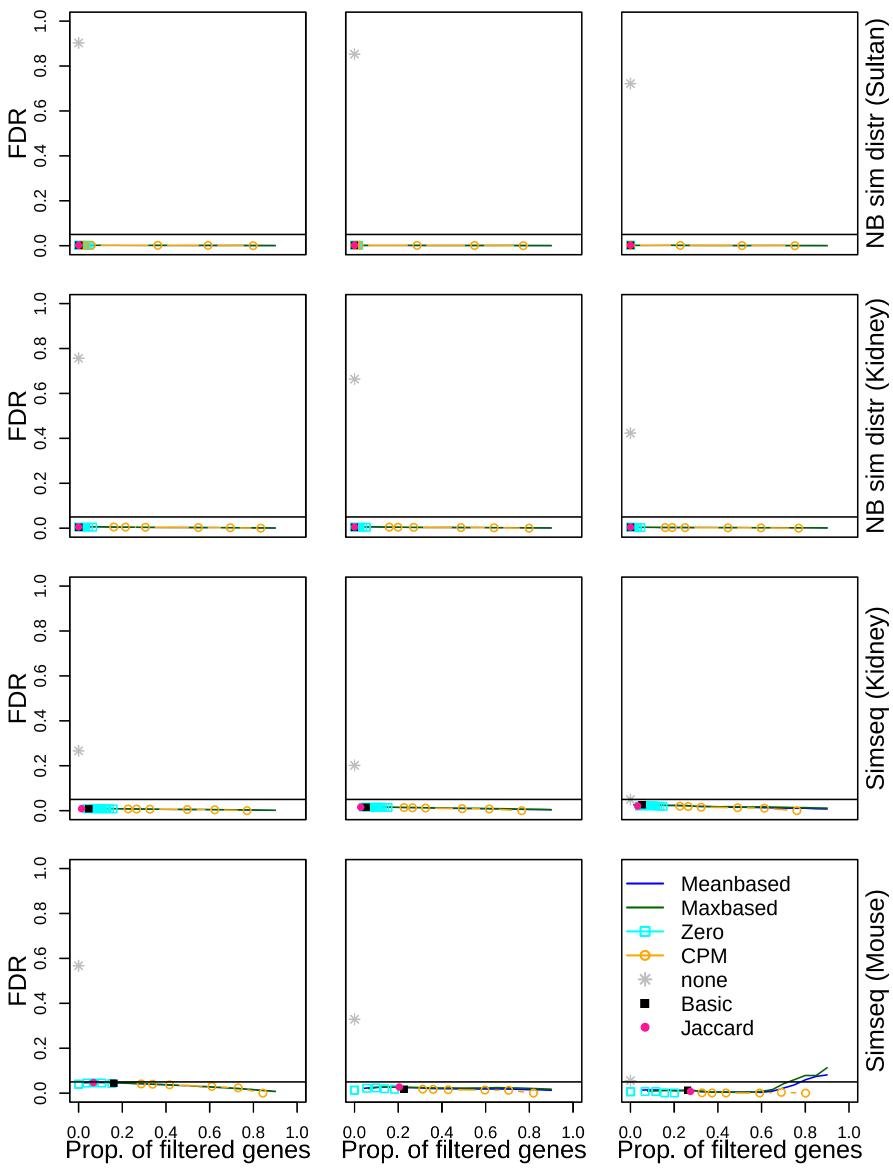

Figure 7: FDR values and percentile thresholds for result of Figure 2 from additional file 2, for several filtering methods for $\pi_0 \in \{0.5, 0.8, 0.99\}$ (from right to left), $m = 10000$, $n_1 = n_2 = 10$, and $\alpha = 0.05$ for several simulation strategies (lfdr adjustment). The FDR of each filtering method is plotted as a function of the actual mean proportion of filtered genes across all simulation runs for the set of genes with at least one non-zero count, only the proportion of the basic filter is based on the total number of hypotheses m. The basic, Jaccard and no filter results are represented by a point because these methods are based on a fixed threshold.

2.3 Adaptive filter: FDR values for $l = 0$ and $l = 5$

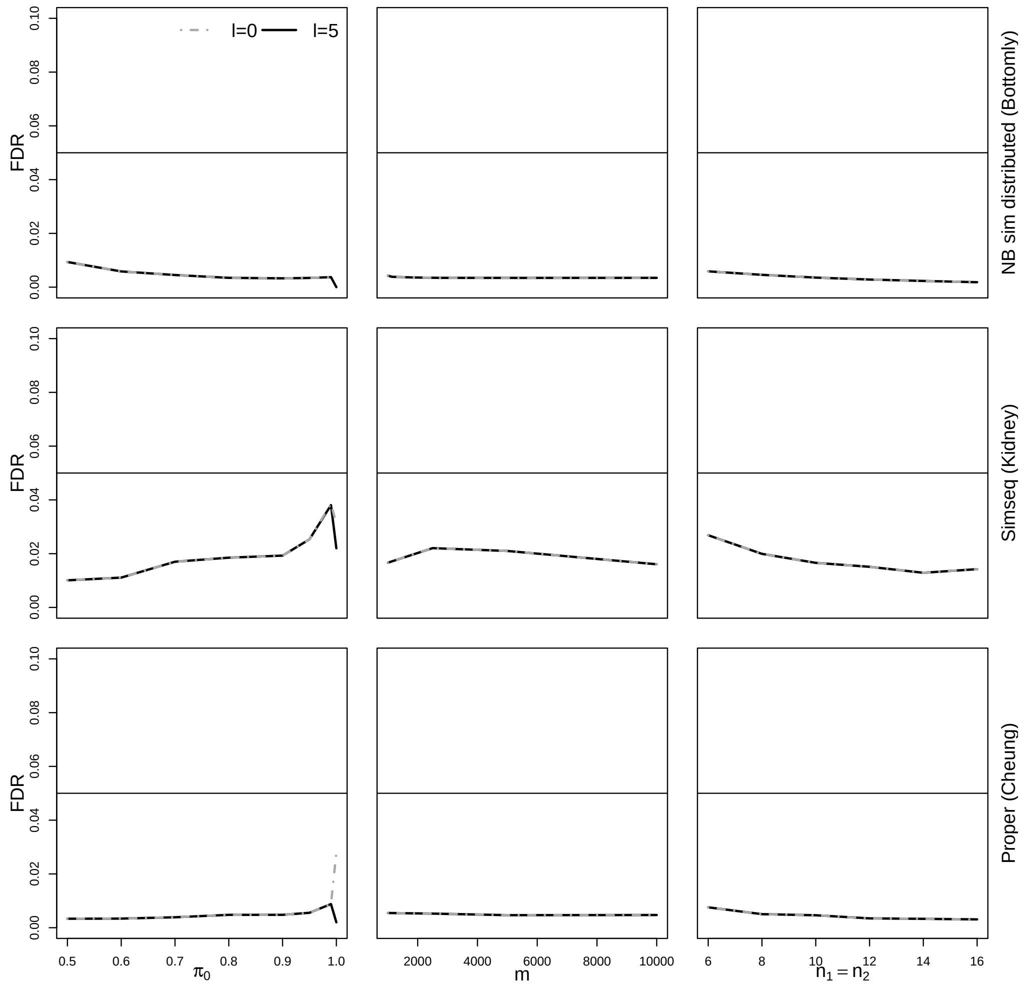

Figure 8: Adaptive filter I: FDR for results of Figure 3 from the manuscript. Simulated FDR for adaptive filter for NB sim distributed (Bottomly data), SimSeq simulation (Kidney data) and PROPER simulation (Cheung data) for varying π_0, m, or $n_1 = n_2$, respectively. $\pi_0 = 0.8$, $m = 10000$, $n_1 = n_2 = 10$ or π_0, m, and $n_1 = n_2$ are parameters on the x-axis, $\alpha = 0.05$ (lfdr adjustment).

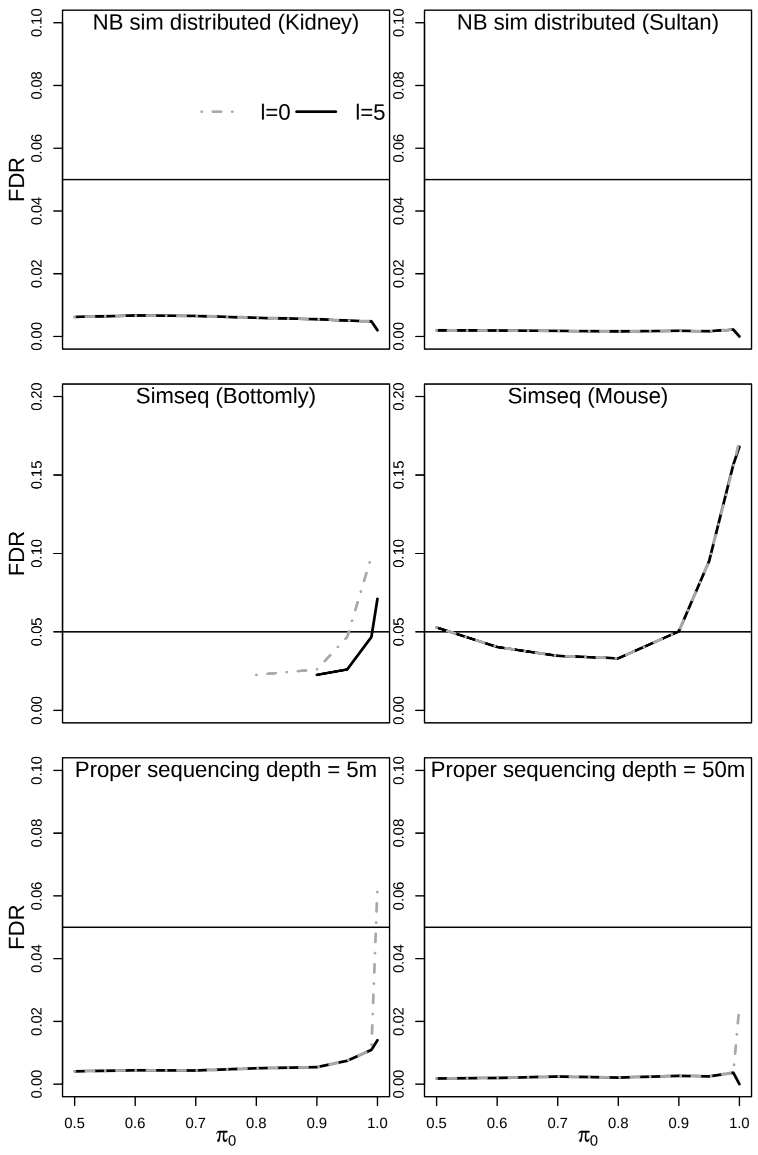

Figure 9: Adaptive filter II: FDR for results of Figure 4 from the manuscript. Simulated FDR for adaptive filter NB sim distributed for Kidney and Sultan data, SimSeq simulation for Bottomly and mouse mammary data sets and PROPER simulation for sequencing depths $5m$ and $50m$ for varying π_0, $m = 10000$, $n_1 = n_2 = 10$ ($n_1 = n_2 = 5$ for the SimSeq Bottomly data and $n_1 = n_2 = 3$ for SimSeq mouse data) and $\alpha = 0.05$ (lfdr adjustment).

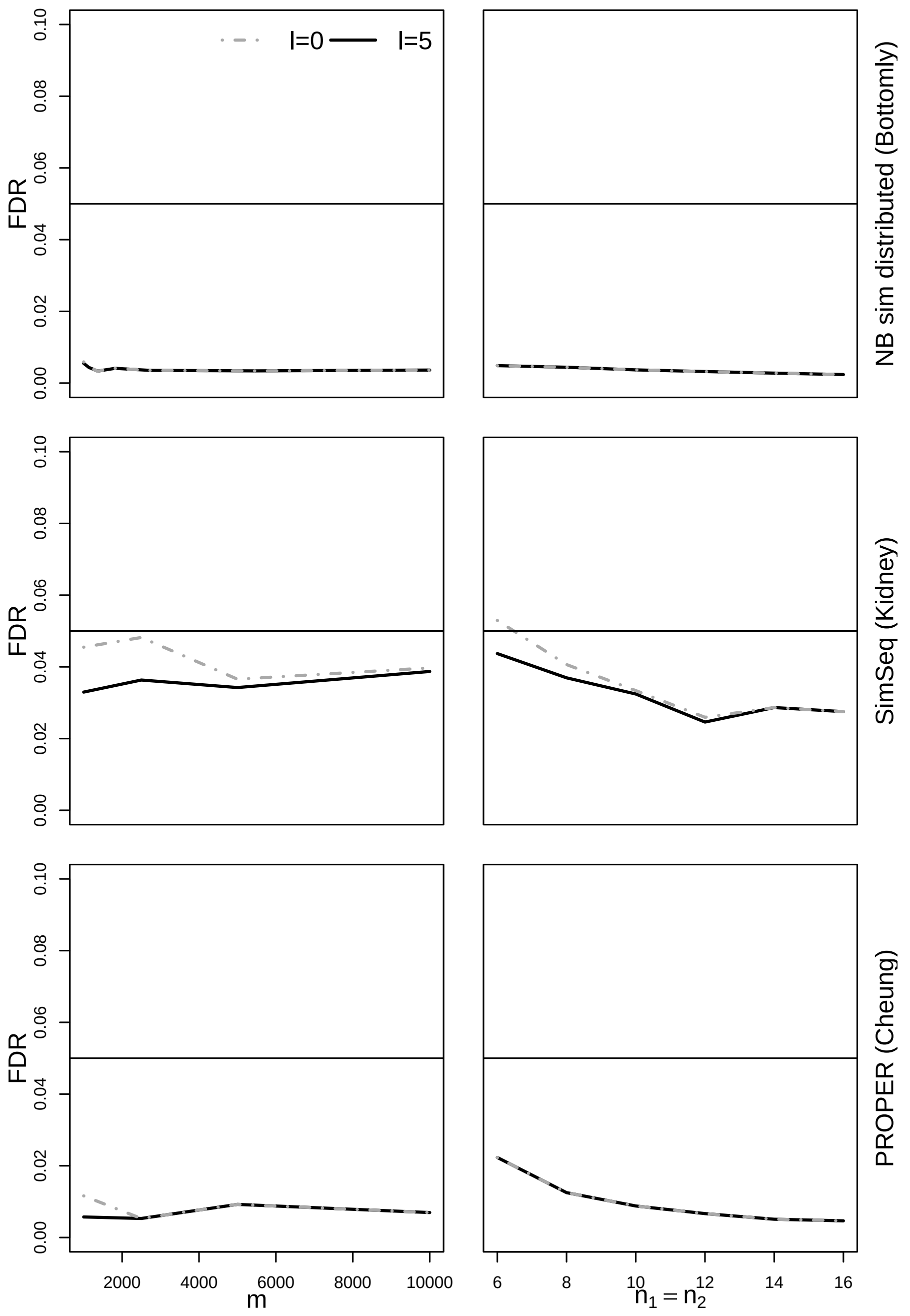

Figure 10: FDR for adaptive filter for results of Figure 3 from additional file 2 for NB sim distributed (Bottomly data set), SimSeq simulation (Kidney data set) and PROPER simulation (Cheung data) for varying m, or $n_1 = n_2$, respectively. If not a parameter on the x-axis, $\pi_0 = 0.99$, $m = 10000$, $n_1 = n_2 = 10$, and $\alpha = 0.05$ (lfdr adjustment).

2.3.1 Influence of filter parameter l and number of hypotheses m on FDR values

Figure 11 shows the simulated FDR of the NB simulation with distributed parameters from the Bottomly data set, the Simseq simulations according to the Kidney data set and the PROPER simulation for $\pi_0 = \{0.8, 0.99, 1\}$, $m = \{100, 500, 1000, 2500, 5000, 10000\}$, $n_1 = n_2 = 10$. For the test decision, each gene with local false discovery rate smaller than $\alpha = 0.05$ is rejected. The simulated FDR of the adaptive filter is given for filter parameters $l = 0, 1, 2, 3, 4, 5$, which means that the adaptive filter can be determined if at least l hypotheses are rejected. If not, the Jaccard filter (reference filter for the simulation study) is chosen.

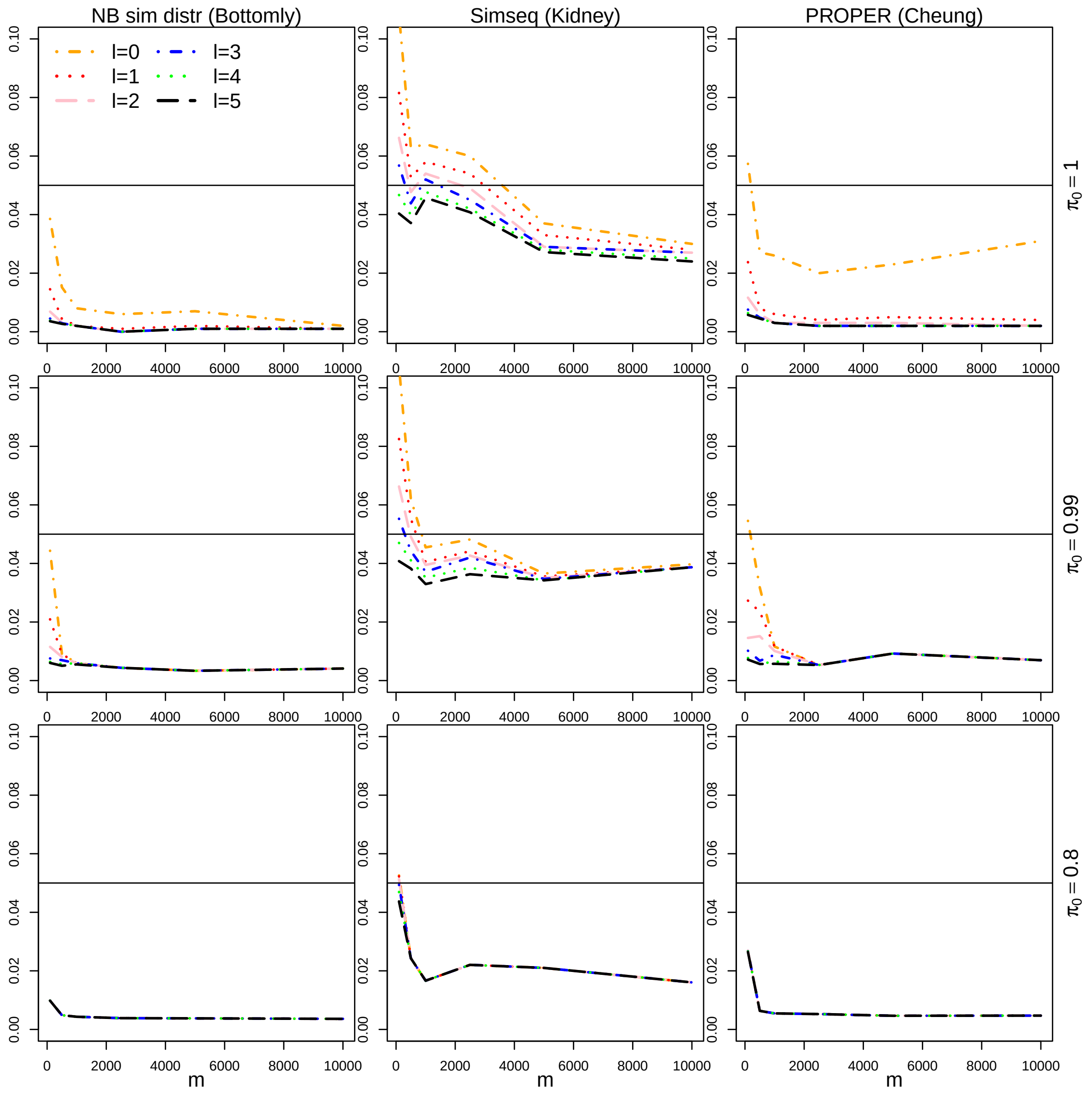

Figure 11: Simulated FDR of NB distributed simulation according to Bottomly, SimSeq simulation according to Kidney data set, and PROPER simulation for $n_1 = n_2 = 10$, $\alpha = 0.05$. Control for multiple testing is performed by the lfdr method.

3 Distribution of lfdr in real data sets

Fig. 12 shows the distribution of local false discovery rates of rejected genes in real data sets recalculated in our manuscript (data sets Bottomly, Sultan, Airway, Mouse, Kidney and Kidney 2).

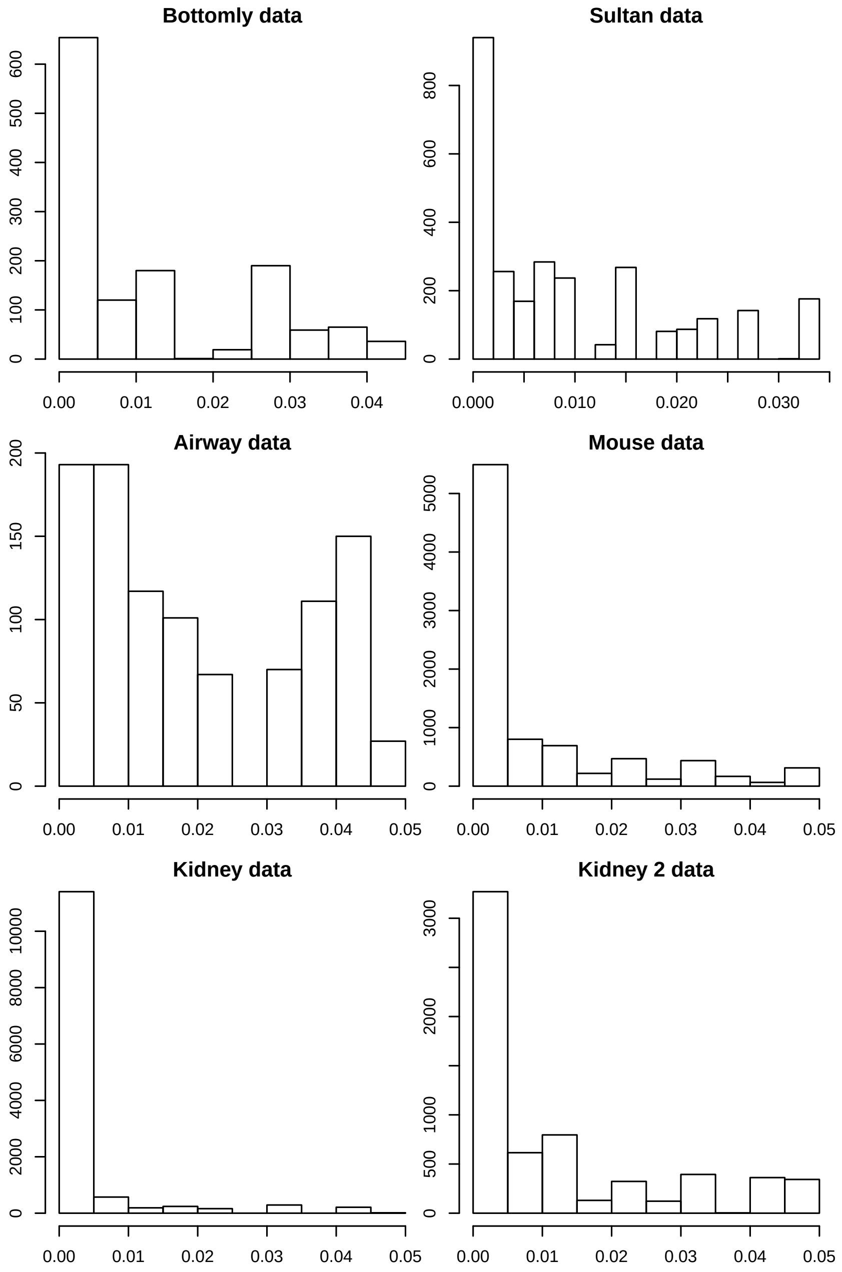

Figure 12: Local False Discovery Rates of rejected genes in real data sets.

4 Power comparison for Benjamini-Hochberg procedure

4.1 Comparison of individual filter strategies

Figures 13 and 16 illustrate the presented simulation scenarios with the same parameters as in Figures 1 and 3 in the manuscript. However, instead of calculating local false discovery rates (lfdr) for each gene and rejecting genes with a lfdr smaller than $\alpha = 0.05$, the Benjamini-Hochberg procedure (Benjamini and Hochberg, 1995) is applied to adjust for multiplicity at significance level α.

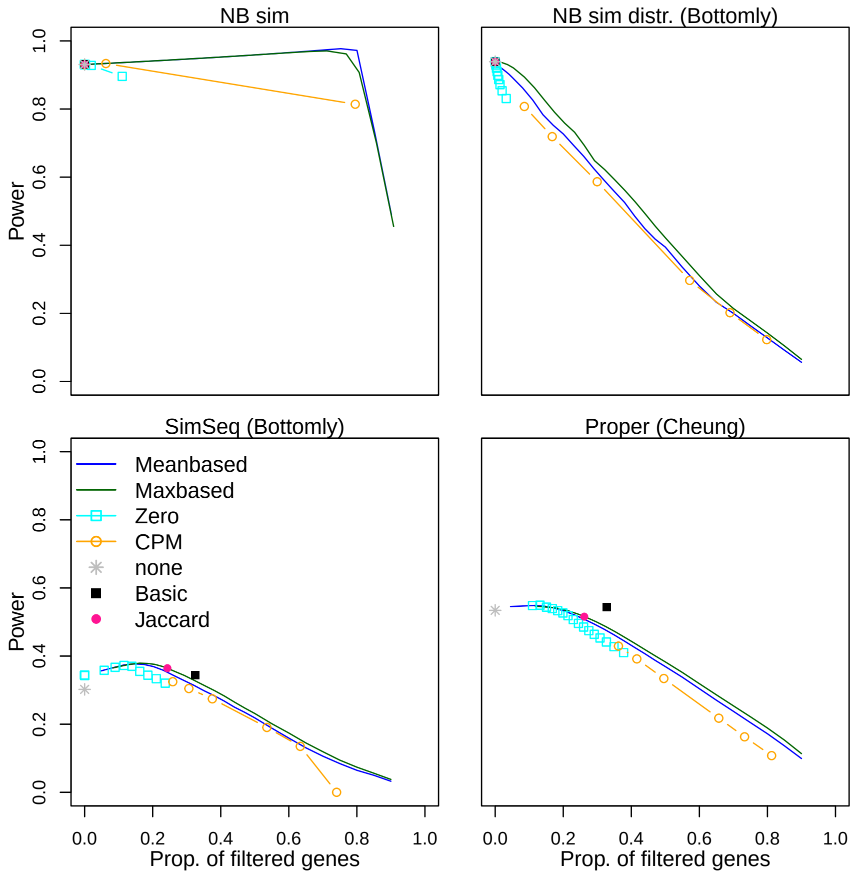

Figure 13: Power comparison of different filters and multiplicity adjustment with the BH procedure. Power values for several filtering methods and simulation strategies for $\alpha = 0.05$, $\pi_0 = 0.8$, $m = 10000$, $n_1 = n_2 = 10$ or $n_1 = n_2 = 5$ for SimSeq (Bottomly), respectively. The power of each filtering method is plotted as a function of the actual mean proportion of filtered genes across all simulation runs for the set of genes with at least one non-zero count, only the percentile of the basic filter is based on the total number of hypotheses m. The basic, Jaccard and no filter resuls are represented by a point because these methods are based on a fixed threshold.

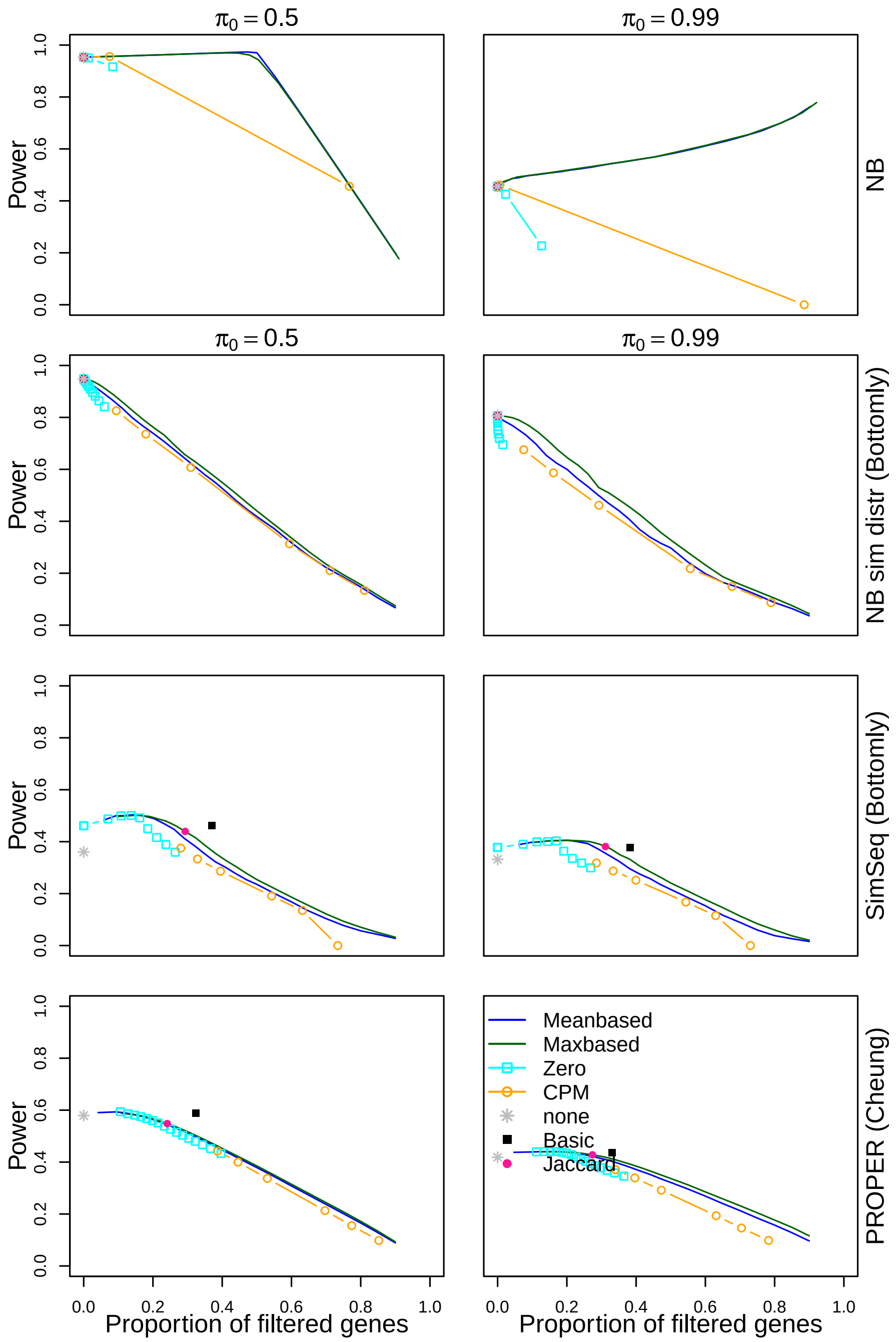

Figure 14: Power comparison of different filters and multiplicity adjustment with the BH procedure, extension of Fig. 13 from additional file 2 for $\pi_0 = 0.5$ and 0.99. Power values for several filtering methods and simulation strategies for $\alpha = 0.05$, $\pi_0 = 0.5, 0.99$, $m = 10000$, $n_1 = n_2 = 10$ or $n_1 = n_2 = 5$ for SimSeq (Bottomly), respectively. The power of each filtering method is plotted as a function of the actual mean proportion of filtered genes across all simulation runs for the set of genes with at least one non-zero count, only the percentile of the basic filter is based on the total number of hypotheses m. The basic, Jaccard and no filter resuls are represented by a point because these methods are based on a fixed threshold.

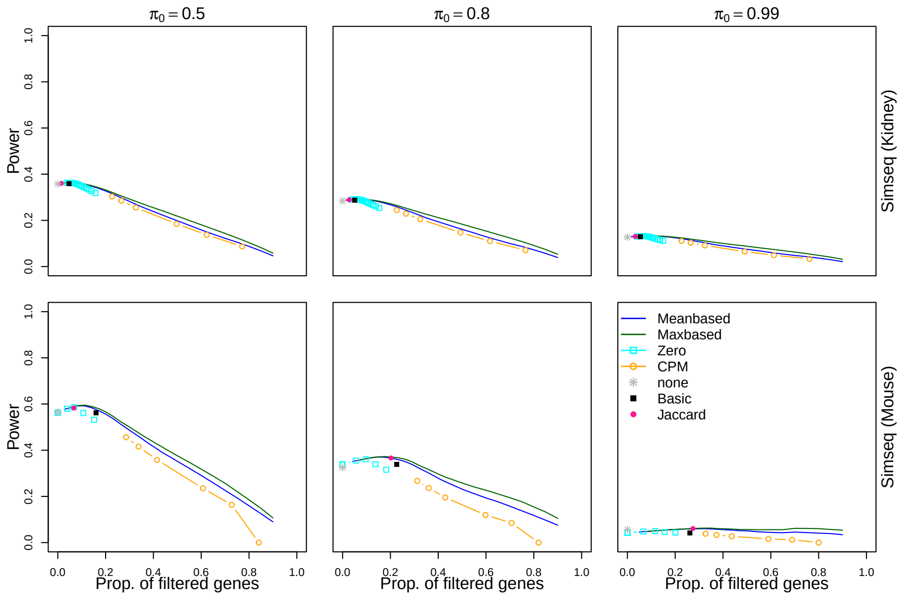

Figure 15: Power comparison of different filters and multiplicity adjustment with the BH procedure. Power values for several filtering methods and simulation strategies for $\alpha = 0.05$, $\pi_0 = 0.5, 0.8, 0.99$, $m = 10000$, $n_1 = n_2 = 10$ or $n_1 = n_2 = 5$ for SimSeq simulation, mouse and Kidney data set, respectively. The power of each filtering method is plotted as a function of the actual mean proportion of filtered genes across all simulation runs for the set of genes with at least one non-zero count, only the percentile of the basic filter is based on the total number of hypotheses m. The basic, Jaccard and no filter resuls are represented by a point because these methods are based on a fixed threshold.

4.2 Adaptive filter

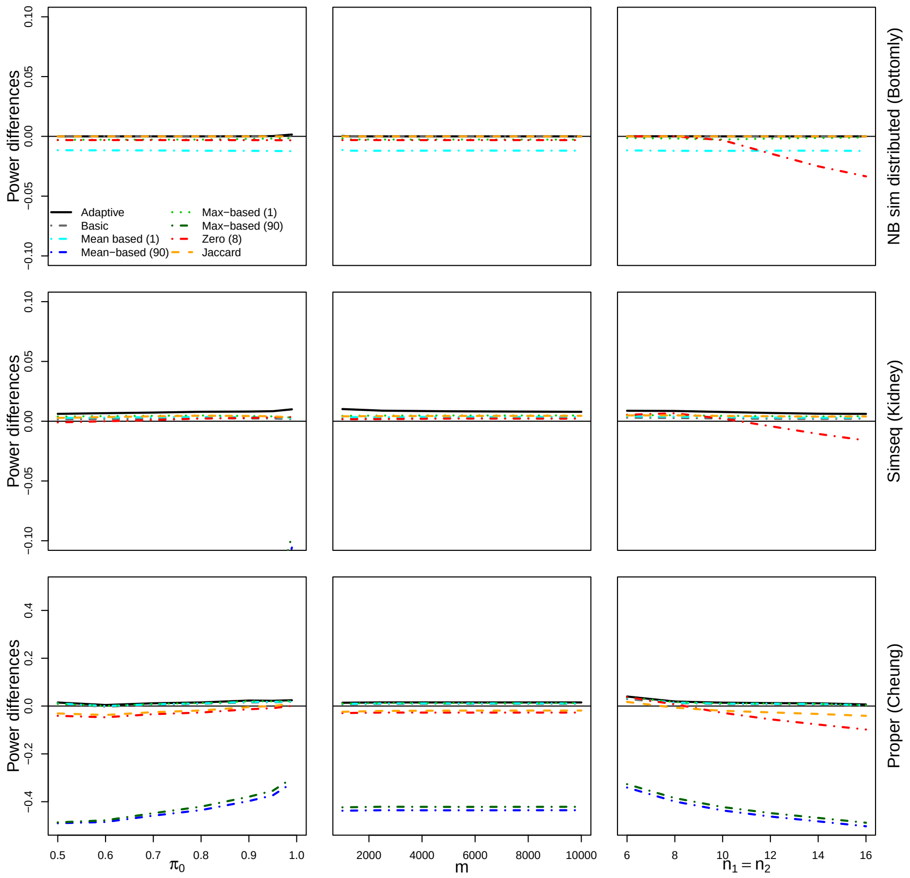

Figure 16: Adaptive filter for multiplicity adjustment with the BH procedure. Differences in power for adaptive filter and selection of applied filter compared to no filter for NB sim distributed (Bottomly data set), SimSeq simulation (Kidney data set) and PROPER simulation (Cheung data) for $\alpha = 0.05$, varying π_0, m, or $n_1 = n_2$, respectively. The plotted filtering methods and the corresponding percentile percentage are given in the legend. If not a parameter on the x-axis, $\pi_0 = 0.8$, $m = 10000$, $n_1 = n_2 = 10$. Note that the range of the y-axis is chosen result-based, on some plots, filtering methods with low power may not be visible.

5 Simulated FDR values for BH adjustment

5.1 Individual filter strategies

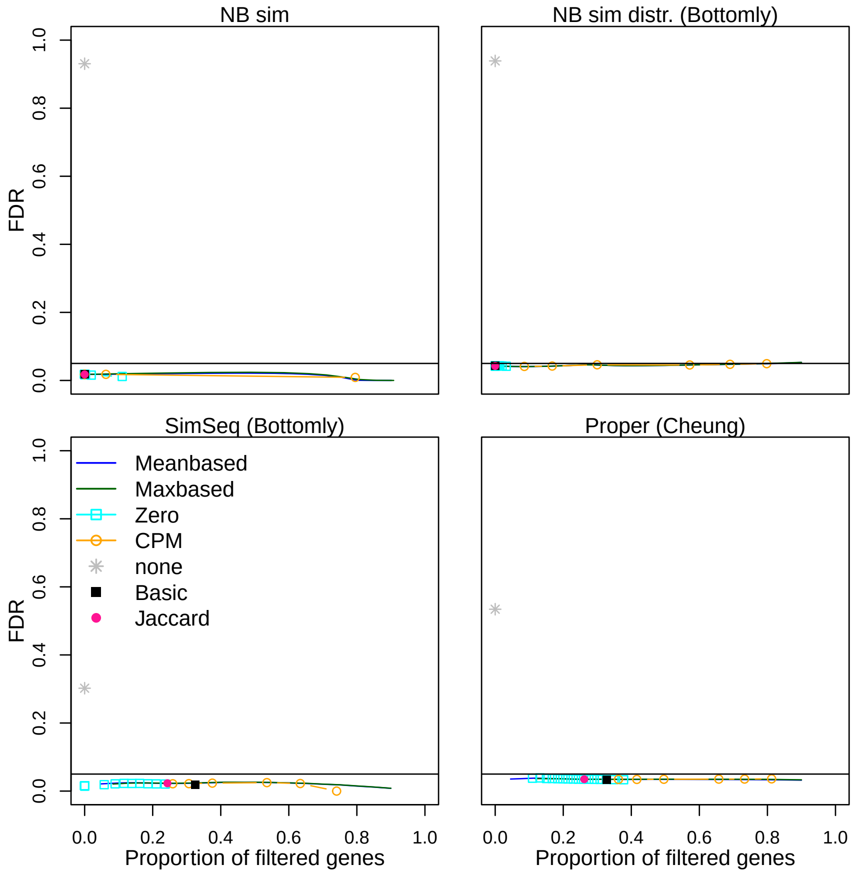

Figure 17: FDR of different filters and multiplicity adjustment with the BH procedure. FDR values for several filtering methods and simulation strategies for $\alpha = 0.05$, $\pi_0 = 0.8$, $m = 10000$, $n_1 = n_2 = 10$ or $n_1 = n_2 = 5$ for SimSeq (Bottomly), respectively. The FDR of each filtering method is plotted as a function of the actual mean proportion of filtered genes across all simulation runs for the set of genes with at least one non-zero count, only the percentile of the basic filter is based on the total number of hypotheses m. The basic, Jaccard and no filter resuls are represented by a point because these methods are based on a fixed threshold.

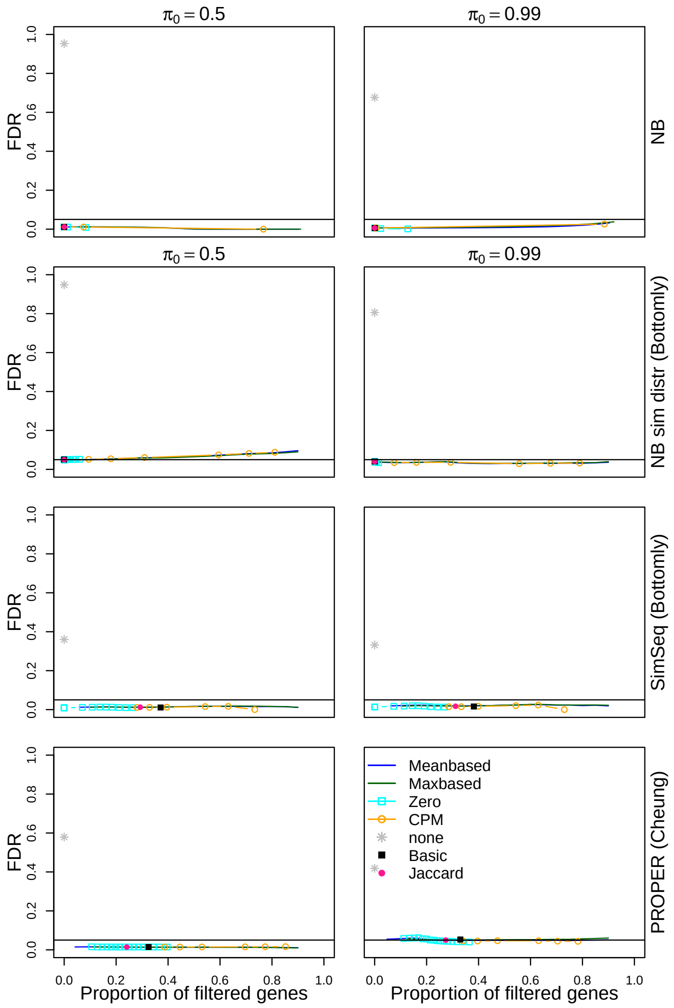

Figure 18: FDR of different filters for BH procedure, extension of Figure 17 from additional file 2 for $\pi_0 = 0.5$ and 0.99: FDR for several filters and simulation strategies for $\alpha = 0.05$, $m = 10000$, $n_1 = n_2 = 10$ (or $n_1 = n_2 = 5$ for SimSeq (Bottomly)). The FDR of each filtering method is plotted as a function of the actual mean proportion of filtered genes across all simulation runs for the set of genes with at least one non-zero count, only the proportion of the basic filter is based on the total number of hypotheses m. The basic, Jaccard and no filter results are represented by a point because these methods are based on a fixed threshold. For the Bottomly data simulation, $\pi_0 \in \{0.9, 0.99\}$.

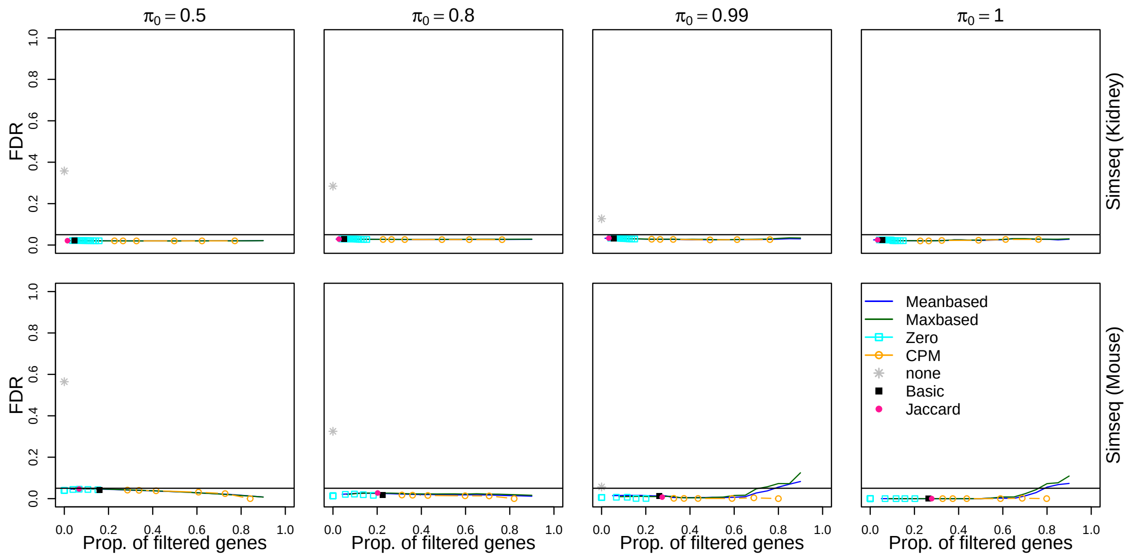

Figure 19: FDR of different filters and multiplicity adjustment with the BH procedure. FDR for several filtering methods and simulation strategies for $\alpha = 0.05$, $\pi_0 = 0.5, 0.8, 0.99, 1$, $m = 10000$, $n_1 = n_2 = 10$ or $n_1 = n_2 = 5$ for SimSeq simulation with mouse and Kidney data sets, respectively. The FDR of each filtering method is plotted as a function of the actual mean proportion of filtered genes across all simulation runs for the set of genes with at least one non-zero count, only the percentile of the basic filter is based on the total number of hypotheses m. The basic, Jaccard and no filter resuls are represented by a point because these methods are based on a fixed threshold.

5.2 Adaptive filter: FDR values for $l = 0$ and $l = 5$

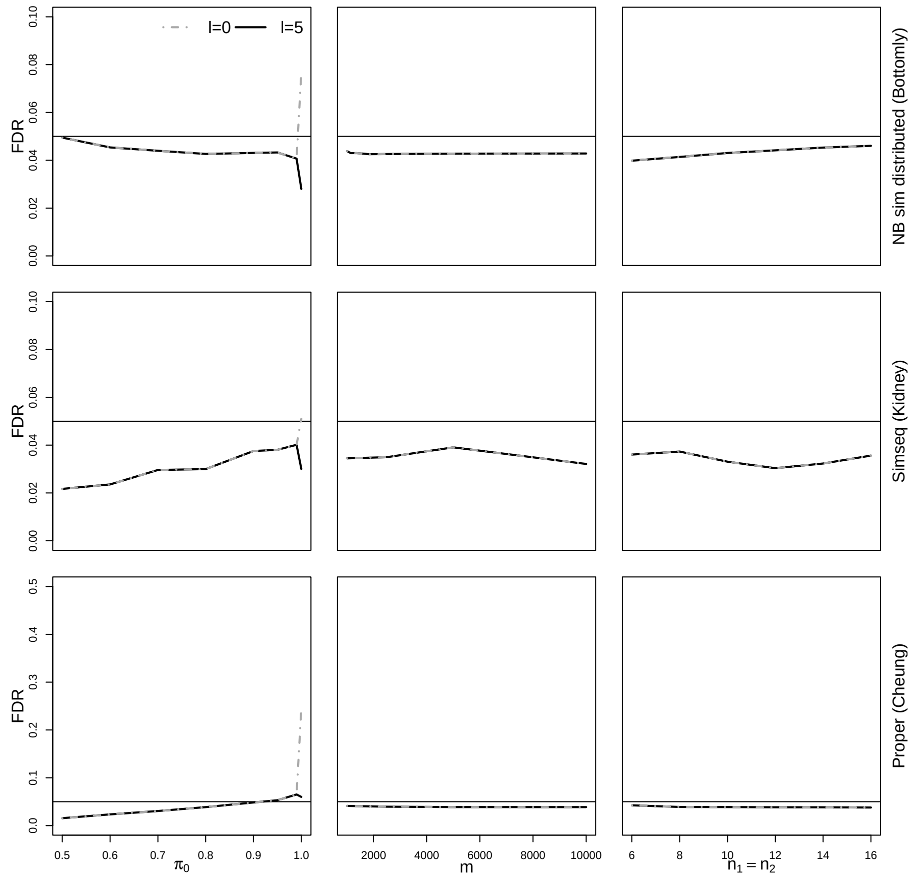

Figure 20: Adaptive filter for multiplicity adjustment with the BH procedure. FDR for adaptive filter for NB sim distributed (Bottomly data set), SimSeq simulation (Kidney data set) and PROPER simulation (Cheung data) for $\alpha = 0.05$, varying π_0, m, or $n_1 = n_2$, respectively. If not a parameter on the x-axis, $\pi_0 = 0.8$, $m = 10000$, $n_1 = n_2 = 10$.

5.2.1 Influence of filter parameter l and number of hypotheses m on FDR values

Figure 21 shows the simulated FDR of the NB simulation with distributed parameters from the Bottomly data set, the Simseq simulations according to the Kidney data set and the PROPER simulation $\pi_0 = \{0.8, 0.99, 1\}$, $n_1 = n_2 = 10$, $\alpha = 0.05$ for the BH adjustment and $m = \{100, 500, 1000, 2500, 5000, 10000\}$. The simulated FDR of the adaptive filter is given for several values for the filter parameter l, which means that the adaptive filter can be determined if at least l hypotheses are rejected. If not, the Jaccard filter (reference filter for the simulation study) is chosen. Note that in some cases, the simulated FDR of individual filtering strategies is already inflated and larger than 0.05, thus also the FDR of the adaptive filter is increased.

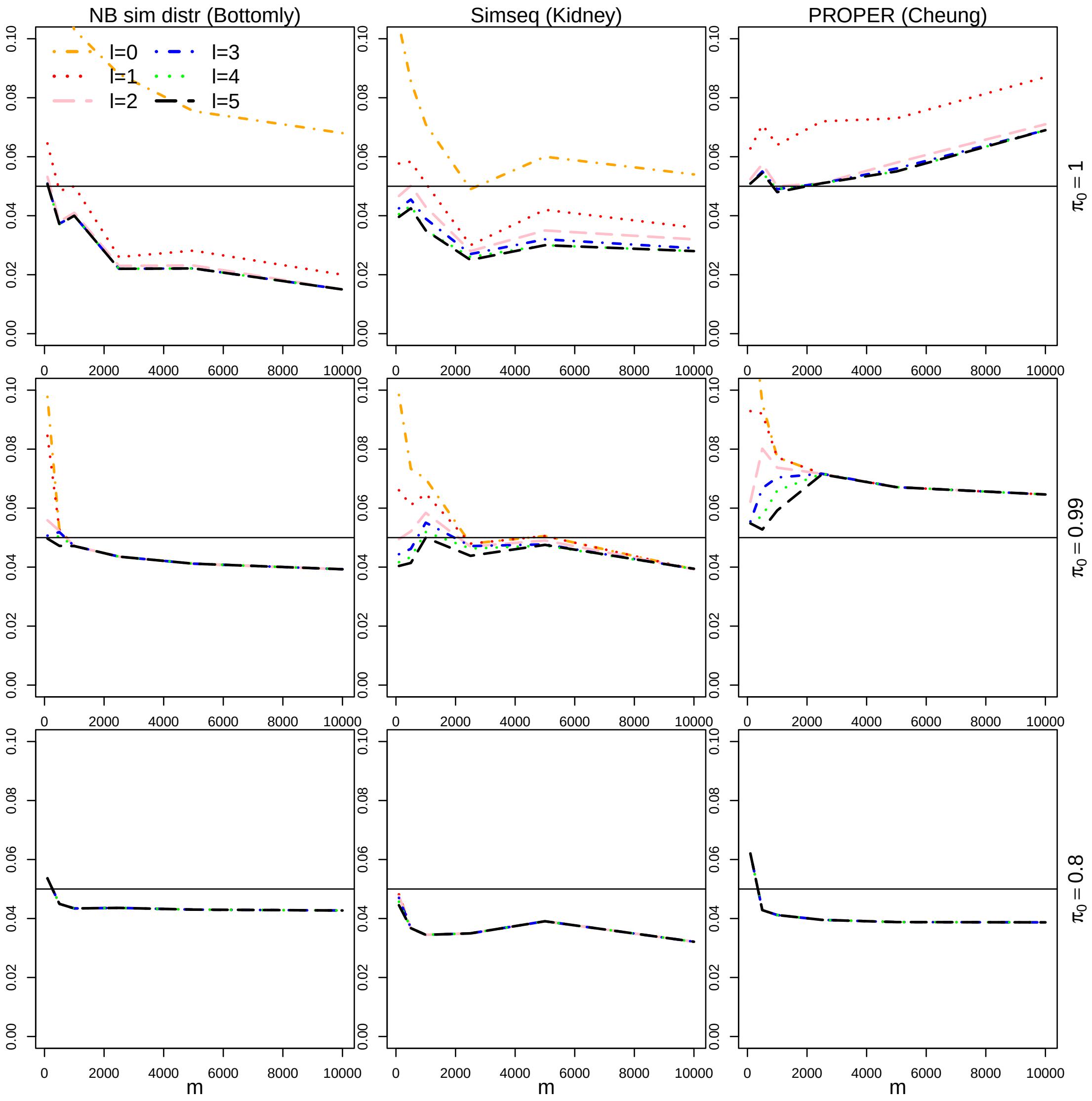

Figure 21: Simulated FDR of NB distributed simulation according to Bottomly, SimSeq simulation according to Kidney data set, and PROPER simulation for $n_1 = n_2 = 10$, $\alpha = 0.05$. Control for multiple testing is performed by the BH procedure.

6 Comparison of individual filter strategies: Modified order of data processing

Figures. 22 and 23 show results of a modified order of data preprocessing for lfdr adjustment. In the first step the raw data are filtered, then data are normalized and finally data analysis is performed (as described as data order (b) in Methods Section in the manuscript).

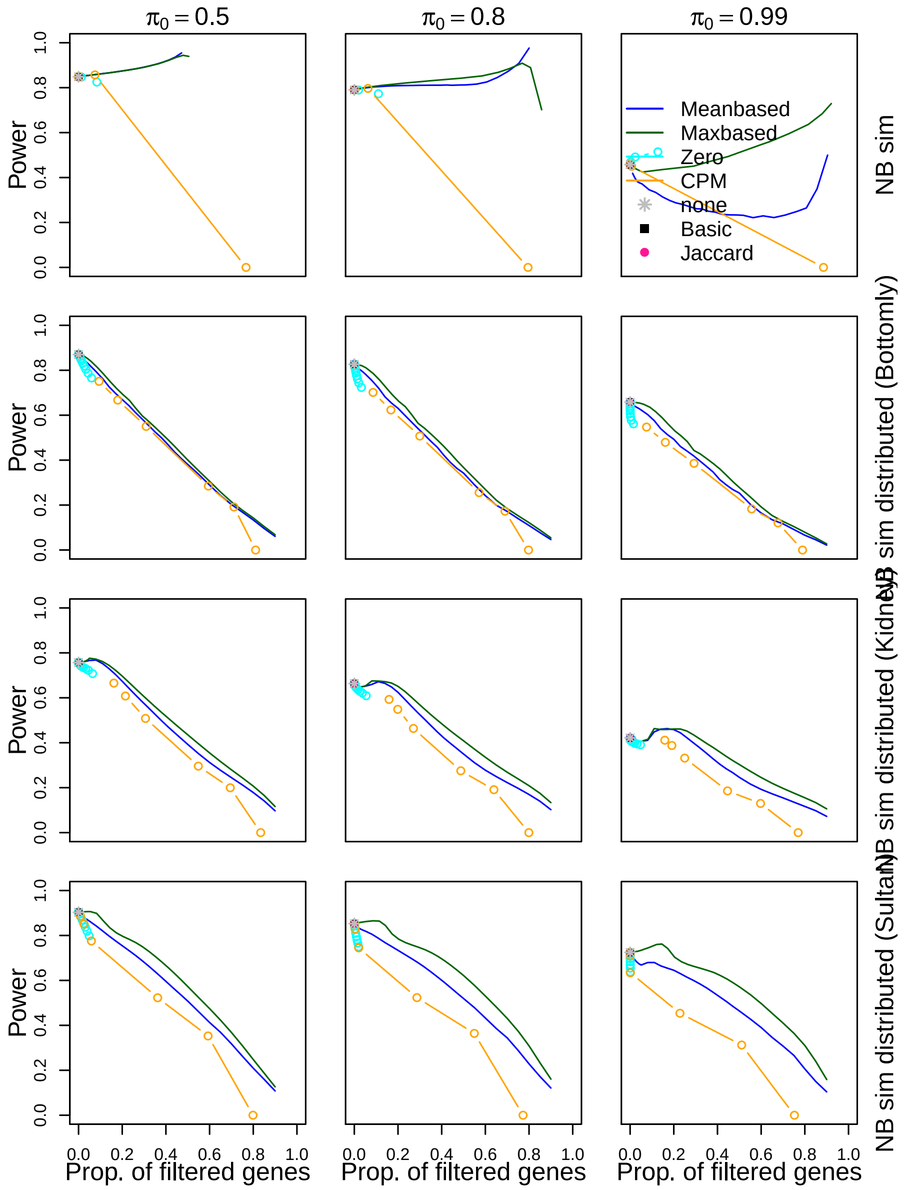

Figure 22: Modified order of data preprocessing: Power values as a function of the proportion of filtered genes for several filtering methods for $\pi_0 = \{0.5, 0.8, 0.99\}$, $m = 10000$, $n_1 = n_2 = 10$, and $\alpha = 0.05$. The basic and Jaccard filter are represented by a point because these methods are based an a single threshold.

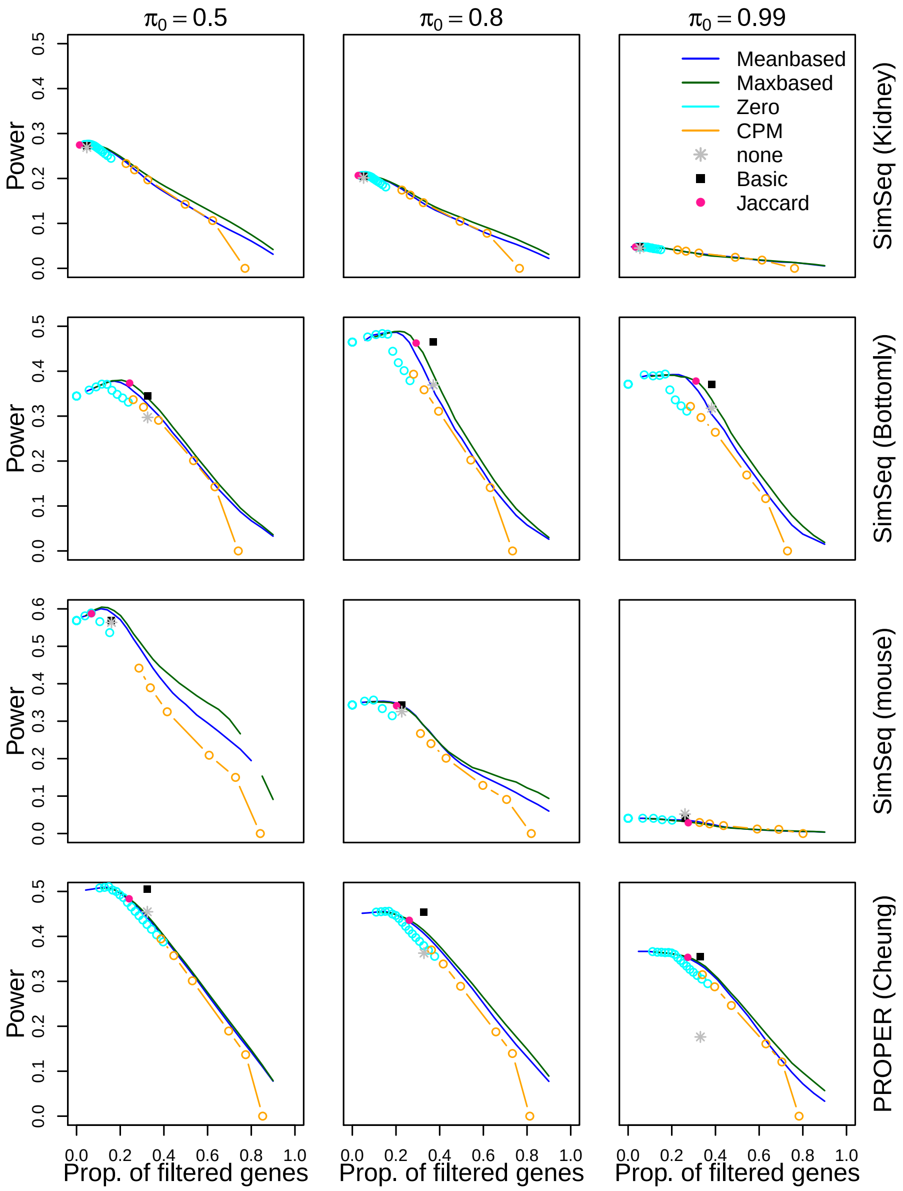

Figure 23: Modified order of data preprocessing: Power values and proportion of filtered genes for data based simulation (SimSeq and PROPER) for several filtering methods for $\alpha = 0.05$, $\pi_0 = \{0.5, 0.8, 0.99\}$, $m = 10000$, $n_1 = n_2 = 10$. For the SimSeq simulation with Bottomly data (row 2), $\pi_0 = \{0.9, 0.95, 0.99\}$.

7 Real data application

7.1 Modified order of data preprocessing

Table 1: Real data application with modfied order of data preprocessing. Maximum number of rejections for each filtering method and the corresponding observed proportion of filtered genes (based on the non-zero genes for the mean, max, zero-based and Jaccard filter) for several data sets, $\alpha = 0.05$. Filtering is performed at the very beginning (order (b)). The adaptive filter with the highest number of rejections is highlighted in bold.

	No filter	Basic	Mean-based	Max-based	Zero-based	Jaccard
Bottomly	1443	1324 (35)	1466 (29)	1516 (27)	1470 (15)	**1539 (34)**
Sultan	-	2801 (83)	3455 (16)	**3544 (15)**	3352 (14)	2827 (42)
Airway	0	1029 (48)	**1634 (60)**	1527 (60)	1220 (39)	1543 (57)
Mouse	9151	8772 (21)	9211 (12)	**9347 (18)**	9092 (11)	8708 (28)
Kidney	**13076**	13075 (3)	**13287 (3)**	13281 (5)	11798 (19)	13265 (2)
Kidney 2	5777	6357 (5)	6356 (8)	3648 (3)	**6357 (7)**	6348 (3)
Yuan						
gill vs. mantle	0	7932 (6)	10993 (47)	**11317 (60)**	8405 (25)	7932 (0)
gill vs. foot	0	7534 (8)	9460 (44)	**10131 (60)**	7647 (27)	7534 (0)
gill vs. vmass	0	6093 (4)	9740 (55)	**10924 (55)**	6131 (24)	6093 (4)
mantle vs. foot	0	**5291 (12)**	4301 (50)	4584 (70)	**5291 (12)**	**5291 (12)**
mantle vs. vmass	0	2468 (6)	2661 (41)	**3178 (74)**	2468 (6)	2468 (6)
foot vs. vmass	0	3605 (7)	4197 (41)	**4429 (50)**	4008 (27)	3605 (7)

7.2 BH procedure

Table 2: Real data application. BH methods. Maximum number of rejections for each filtering method and the corresponding observed proportion of filtered genes in parentheses (for the basic filter based on all genes, for other filters on the non-zero genes) for several data sets, $\alpha < 0.05$. Filtering is performed at the end (order (a)). The adaptive filter with the highest number of rejections is highlighted in bold.

	No filter	Basic	Mean-based	Max-based	Zero-based	Jaccard
Bottomly	1843	1915 (35)	**2082 (20)**	**2081 (22)**	2067 (18)	1945 (34)
Sultan	-	3556 (89)	3662 (16)	3647 (15)	**3684 14)**	3041 (42)
Airway	1542	1843 (48)	1918 (55)	1957 (60)	1778 (36)	**1961 (57)**
Airway 2	-	170 (52)	385 (65)	**421 (70)**	208 (25)	360 (54)
Mouse	9943	10072 (21)	**10179 (8)**	10161 (7)	10171 (5)	9321 (28)
Kidney	14040	14066 (3)	**14082 (2)**	14075 (2)	12386 (19)	14075 (2)
Kidney 2	7154	7159 (5)	7257 (5)	7250 (5)	**7270 (5)**	7214 (3)
Yuen						
gill vs. mantle	11349	11349 (6)	12856 (32)	**12860 (35)**	12003 (25)	11349 (0)
gill vs. foot	10704	10704 (8)	**12143 (33)**	12100 (36)	11255 (14)	10704 (0)
gill vs. vmass	8269	8269 (4)	10015 (38)	**10075 (41)**	9139 (23)	8269 (0)
mantle vs. foot	7547	7547 (12)	**8369 (33)**	8358 (34)	7829 (13)	7547 (0)
mantle vs. vmass	3943	3943 (6)	4913 (47)	**5163 (60)**	4436 (23)	3943 (0)
foot vs. vmass	5270	5270 (7)	6628 (39)	**6788 (56)**	6078 (27)	5270 (0)

References

Y. Benjamini and Y. Hochberg (1995) Controlling the False Discovery Rate: A Practical and Powerful Approach to Multiple Testing *J.R.Statist.Soc.B* **57**, 289-300.
