# Supplementary material for: Impact of adaptive filtering on power and false discovery rate in RNA-seq experiments
Source: BMC Bioinformatics. 2022 Sep 24;23:388. doi: 10.1186/s12859-022-04928-z (PMC9509565; doi:10.1186/s12859-022-04928-z)
Supplement: Supplementary file 3 — Additional file 3. Data example for the R-code from Additional file 4. [file 12859_2022_4928_MOESM3_ESM.pdf]

Impact of adaptive filtering on power and False Discovery Rate in RNA-seq experiments Additional file 3

Sonja Zehetmayer, Martin Posch, Alexandra Graf

February 2, 2022

1 Data example for example programming code in additional file 4

Example programming code in the R programming language (http://cran.r-project.org/) for the manuscript ”Impact of adaptive filtering on power and False Discovery Rate in RNA-seq experiments” is provided in ”Additionalfile4.R”. Details on the procedure are given in the manuscript.

Mainfunction: *adaptive.filter.func*: This function applies several filters as basic filter, mean-based filter, max-based filter, zero-based, and Jaccard filter for a matrix of input count data. It then searches the adaptive filter, which is defined as the filter leading to the largest number of rejections. First all genes with only zero counts are deleted (basic filter) and normalized with TMM. Data analysis with voom and limma is performed. Then all filters and corresponding thresholds are applied and the adaptive filter is determined. If the number of rejections, however, is smaller than the filter parameter l, a pre-defined reference filter (in this example code the Jaccard filter) has to be applied. For mean-based and max-based filters percentiles have to be defined, for Zero-based filter thresholds have to be defined.

Input parameters of main function *adaptive.filter.func*:

- counts: count data (as matrix or dataframe)
- groupvar: vector denoting the two condition groups
- meanperc: percentiles for mean-based filter, scalar or vector
- maxperc: percentiles for max-based filter, scalar or vector
- zerothresh: threshold for zero-based filter, scalar or vector
- l: chosen filter parameter
- alpha: significance threshold (FDR level)

- method:
  - BH: Benjamini-Hochberg adjustment
  - lfdr: local false discovery rates adjustment

Output parameters of main function *adaptive.filter.func*: A list of the following parameters is generated:

- adaptive.filter: name of adaptive filter
- n.rej.adaptive.filter: number of rejections of adaptive filter
- percentiles: actual percentiles of all considered filters
- n.rej: numbers of rejections of all considered filters
- rej: rejection matrix of adaptive filter ('TRUE'=rejected, 'FALSE'=not rejected or 'NA'=removed by adaptive filter)

1.1 Example

We show an example of the Kidney data set from the SimSeq R-package. To decrease runtime, a selection of 10 samples per group is made.

```
library(SimSeq)
data(kidney) #load Kidney data set
#a selection of 10 samples per group is made:
a<-c(95,  104,  89,  92,  73,  83, 4,  65,  17, 118)
b<-c(110,  78,  34,  58,  70, 117,  30,  35, 109,  53)
selection<-sort(c(a,b))

# Matrix of read counts from KIRC dataset:
counts <- kidney$counts[,selection]
# Treatment vector indicating Non-Tumor or Tumor columns
groupvar <- kidney$treatment[selection]

#define percentiles/thresholds for max/mean/zero based filter
meanperc<-seq(0.01,0.05,0.01)
maxperc<-seq(0.01,0.05,0.01)
zerothresh<-c(20:15)
```

1.1.1 Benjamini-Hochberg adjustment

The adaptive filter with the largest number of rejections is the zero-based filter with threshold 15 and 5271 rejections.

```
adaptive.filter.func(counts=counts,groupvar=groupvar,meanperc=meanperc,
 maxperc=maxperc,zerothresh=zerothresh,l=5,alpha=0.05,method="BH")
```

```
[[1]]
[1] "Zero-based.15"

[[2]]
[1] 5271

[[3]]
   Basic filter Mean-based.0.01 Mean-based.0.02 Mean-based.0.03 Mean-based.0.04
          0.000           0.014           0.024           0.031           0.040
Mean-based.0.05  Max-based.0.01  Max-based.0.02  Max-based.0.03  Max-based.0.04
          0.051           0.028           0.028           0.044           0.044
 Max-based.0.05   Zero-based.20   Zero-based.19   Zero-based.18   Zero-based.17
          0.054           0.000           0.000           0.020           0.032
  Zero-based.16   Zero-based.15         Jaccard
          0.043           0.051           0.028

[[4]]
   Basic filter Mean-based.0.01 Mean-based.0.02 Mean-based.0.03 Mean-based.0.04
           5168            5193            5207            5222            5232
Mean-based.0.05  Max-based.0.01  Max-based.0.02  Max-based.0.03  Max-based.0.04
           5260            5219            5219            5236            5236
 Max-based.0.05   Zero-based.20   Zero-based.19   Zero-based.18   Zero-based.17
           5270            5168            5168            5196            5225
  Zero-based.16   Zero-based.15         Jaccard
           5235            5271            5219

[[5]]
          ?|100130426           ?|100133144           ?|100134869
                   NA                 FALSE                 FALSE
              ?|10357               ?|10431              ?|155060
                FALSE                  TRUE                 FALSE
              ?|26823              ?|280660              ?|340602
                FALSE                    NA                 FALSE
             ?|388795              ?|390284              ?|391343
                FALSE                 FALSE                    NA
             ?|391714              ?|553137               ?|57714
                FALSE                 FALSE                 FALSE
             ?|645851              ?|652919              ?|653553
                FALSE                  TRUE                 FALSE
             ?|728603              ?|728788              ?|729884
                   NA                 FALSE                 FALSE
               ?|8225               ?|90288                A1BG|1
                FALSE                 FALSE                 FALSE
           A1CF|29974           A2BP1|54715           A2LD1|87769
                FALSE                  TRUE                 FALSE
          A2ML1|144568                 A2M|2          A4GALT|53947
```

```
                FALSE                    FALSE                    FALSE
          A4GNT|51146              AAA1|404744                AAAS|8086
                FALSE                     TRUE                    FALSE
         AACSL|729522               AACS|65985           AADACL2|344752
                FALSE                    FALSE                       NA
       AADACL3|126767           AADACL4|343066                AADAC|13
                   NA                     TRUE                    FALSE
          AADAT|51166              AAGAB|79719               AAK1|22848
                FALSE                     TRUE                     TRUE
              AAMP|14                 AANAT|15              AARS2|57505
                FALSE                    FALSE                     TRUE
         AARSD1|80755                  AARS|16          AASDHPPT|60496
                FALSE                    FALSE                    FALSE
         AASDH|132949               AASS|10157               AATF|26574
                FALSE                     TRUE                     TRUE
            AATK|9625                  ABAT|18             ABCA10|10349
                FALSE                    FALSE                    FALSE
        ABCA11P|79963             ABCA12|26154            ABCA13|154664
                 TRUE                    FALSE                     TRUE
       ABCA17P|650655                 ABCA1|19                 ABCA2|20
                FALSE                     TRUE                    FALSE
             ABCA3|21                 ABCA4|24              ABCA5|23461
                FALSE                     TRUE                    FALSE
...
```

1.1.2 Local false discovery rate adjustment

The adaptive filter with the largest number of rejections is the mean-based filter with percentile 0.03 and 4699 rejections. Note that in this example many other filtering strategies lead to the same number of rejections. In this case, this R-program selects the first one as adaptive filter.

```
adaptive.filter.func(counts=counts,groupvar=groupvar,meanperc=meanperc,
 maxperc=maxperc,zerothresh=zerothresh,l=5,alpha=0.05,method="lfdr")
[[1]]
[1] "Mean-based.0.03"

[[2]]
[1] 4699

[[3]]
   Basic filter Mean-based.0.01 Mean-based.0.02 Mean-based.0.03 Mean-based.0.04
          0.000           0.014           0.024           0.031           0.040
Mean-based.0.05  Max-based.0.01  Max-based.0.02  Max-based.0.03  Max-based.0.04
          0.051           0.028           0.028           0.044           0.044
 Max-based.0.05   Zero-based.20   Zero-based.19   Zero-based.18   Zero-based.17
```

```
          0.054            0.000            0.000            0.020            0.032
 Zero-based.16    Zero-based.15          Jaccard
          0.043            0.051            0.028

[[4]]
  Basic filter Mean-based.0.01 Mean-based.0.02 Mean-based.0.03 Mean-based.0.04
          4398            4653            4653            4699            4699
Mean-based.0.05  Max-based.0.01  Max-based.0.02  Max-based.0.03  Max-based.0.04
          4694            4699            4699            4649            4649
 Max-based.0.05   Zero-based.20   Zero-based.19   Zero-based.18   Zero-based.17
          4693            4398            4398            4653            4653
  Zero-based.16   Zero-based.15         Jaccard
          4699            4699            4699


[[5]]
          ?|100130426           ?|100133144           ?|100134869
                   NA                 FALSE                 FALSE
              ?|10357               ?|10431              ?|155060
                FALSE                  TRUE                 FALSE
              ?|26823              ?|280660              ?|340602
                FALSE                 FALSE                 FALSE
             ?|388795              ?|390284              ?|391343
                FALSE                 FALSE                 FALSE
             ?|391714              ?|553137               ?|57714
                FALSE                 FALSE                 FALSE
             ?|645851              ?|652919              ?|653553
                FALSE                  TRUE                 FALSE
             ?|728603              ?|728788              ?|729884
                FALSE                 FALSE                 FALSE
               ?|8225               ?|90288                A1BG|1
                FALSE                 FALSE                 FALSE
           A1CF|29974           A2BP1|54715           A2LD1|87769
                FALSE                  TRUE                 FALSE
         A2ML1|144568                 A2M|2          A4GALT|53947
                FALSE                 FALSE                 FALSE
          A4GNT|51146          AAA1|404744             AAAS|8086
                FALSE                  TRUE                 FALSE
         AACSL|729522            AACS|65985        AADACL2|344752
                FALSE                 FALSE                    NA
       AADACL3|126767        AADACL4|343066              AADAC|13
                   NA                  TRUE                 FALSE
          AADAT|51166           AAGAB|79719            AAK1|22848
                FALSE                  TRUE                  TRUE
              AAMP|14              AANAT|15           AARS2|57505
                FALSE                 FALSE                  TRUE
          AARSD1|80755               AARS|16        AASDHPPT|60496
```

```
                 FALSE                  FALSE                  FALSE
          AASDH|132949            AASS|10157             AATF|26574
                 FALSE                   TRUE                   TRUE
             AATK|9625                ABAT|18           ABCA10|10349
                 FALSE                  FALSE                  FALSE
        ABCA11P|79963           ABCA12|26154          ABCA13|154664
                 FALSE                  FALSE                   TRUE
       ABCA17P|650655               ABCA1|19               ABCA2|20
                 FALSE                   TRUE                  FALSE
...
```
